# Fate, occurrence, and regional-scale emissions of neonicotinoid pesticides and their metabolites in wastewater treatment plants in suburban Shanghai, China

**DOI:** 10.1016/j.eehl.2026.100215

**Published:** 2026-01-20

**Authors:** Yunhui Zhang, Lite Meng, Wenfei Yu, Yang Wen, Hui Wang, Mengchen Sun, Yuanchen Chen, Bin Dong, Jörg Rinklebe

**Affiliations:** a College of Environmental Science and Engineering, Tongji University, Shanghai 200092, China; b Key Laboratory of Urban Water Supply, Water Saving and Water Environment Governance in the Yangtze River Delta of Ministry of Water Resources, Shanghai 200092, China; c State Key Laboratory of Pollution Control and Resource Reuse, Shanghai 200092, China; d State Key Laboratory of Green Chemical Synthesis and Conversion, Zhejiang Key Laboratory of Clean Energy Conversion and Utilization, Science and Education Integration College of Energy and Carbon Neutralization, Zhejiang University of Technology, Hangzhou 310014, China; e State Key Laboratory for the Quality and Safety of Agro-Products, Institute of Agro-Product Safety and Nutrition, Zhejiang Academy of Agricultural Sciences, Hangzhou 310021, China; f College of Emergency Technology and Management, North China Institute of Science and Technology, Langfang 065201, China; g Guangxi Key Laboratory of Environmental Pollution Control Theory and Technology, Guilin University of Technology, Guilin 541006, China; h School of Architecture and Civil Engineering, Institute of Foundation Engineering, Water and Waste Management, Laboratory of Soil and Groundwater Management, University of Wuppertal, Wuppertal 42097, Germany

**Keywords:** Neonicotinoid pesticides, WWTPs, Metabolites, Migration, Distribution

## Abstract

Neonicotinoid pesticides (NEOs) are emerging contaminants with potential ecological and human health risks. However, their sources, transformation dynamics, and emission pathways in urban wastewater systems remain poorly quantified. This study systematically investigates the spatial distribution, sources, and transformation of 8 parent NEOs (pNEOs) and 6 metabolites (mNEOs) in the influents of 21 wastewater treatment plants (WWTPs) in suburban Shanghai, China. The average concentrations of ΣpNEOs and ΣmNEOs were 568.17 ng/L and 478.20 ng/L, respectively, with significant spatial variations. pNEOs were dominated by nitenpyram (NIT) and dinotefuran (DIN), while mNEOs, such as desnitro-imidacloprid (DN-IMI) and dinotefuran-urea (DIN-U), showed higher abundances. Correlation and cluster analyses reveal pNEOs primarily originate from agricultural activities, whereas mNEOs likely stem from both agricultural and industrial sources, including pesticide production residues. A novel model incorporating Monte Carlo simulations estimates point-source emissions from the 21 WWTPs at 264.57 kg/a for pNEOs and 269.34 kg/a for mNEOs, with total Shanghai-wide emissions reaching 2947.03 kg/a and 1056.56 kg/a, respectively. This study highlights the critical role of WWTPs in discharging NEOs into receiving water bodies, underscoring the need for integrated management strategies targeting agricultural and industrial inputs to WWTPs as well as for the advancement of WWTP processes designed to eliminate emerging contaminants.

## Highlights

• Spatial distribution of pNEOs and mNEOs in influents of 21 WWTPs in suburban Shanghai.
• PNEOs stem mainly from agriculture; mNEOs may be from industrial and agricultural sources.
• A novel emission model was developed to estimate annual emissions of pNEOs and mNEOs from WWTPs.
• Annual emission of pNEOs from all WWTPs in Shanghai was threefold higher than that of mNEOs.

## Graphical abstract

**Figure:**
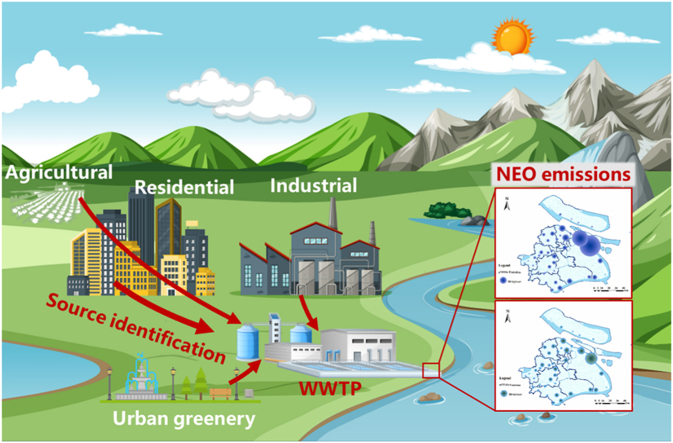

## 1 Introduction

Neonicotinoid pesticides (NEOs), the most widely used class of insecticides globally, have revolutionized pest management in agriculture, urban greenery, and public health due to their systemic activity and high selectivity for insect nicotinic acetylcholine receptors [1,2]. However, their extensive use has raised concerns about environmental persistence, bioaccumulation, and potential risks to non-target organisms, including pollinators, aquatic life, and human health [3]. Parent NEOs (pNEOs) and their metabolites (mNEOs) have been detected in various environmental matrices, such as surface water [4,5], soil [6,7], and drinking water [8,9], reflecting the widespread anthropogenic inputs that increasingly disrupt the natural balance of soil and water systems. While these ecosystems possess inherent self-purification capacities (e.g., microbial degradation of contaminants in soil), the scale of NEO inputs often exceeds such buffering limits. Excess NEOs inhibit beneficial soil microbes and accumulate in aquatic invertebrates, highlighting the need for comprehensive investigations into their fate and transport in anthropogenically pressured urban water systems.

Municipal wastewater treatment plants (WWTPs) serve as critical nodes in urban water cycles by receiving a complex mixture of contaminants from diverse sources, including agricultural runoff, industrial discharges, and domestic sewage [10,11]. Previous studies have reported the presence of NEOs in WWTP influents worldwide, with concentrations often exceeding those in natural water bodies. For example, in China and U.S. WWTPs, pNEOs were detected at ng/L to μg/L levels, indicating significant anthropogenic inputs [[12], [13], [14]]. Conventional treatment processes, however, exhibit variable and often limited removal efficiencies for NEOs (0–68%) [13,15], leading to their discharge into receiving water bodies as persistent point-source pollutants. This is particularly critical in megacities like Shanghai with rapid urbanization, dense population, intensive urban greenery, and partial agriculture and industrial activities, which create a mosaic of NEO exposure pathways.

Despite growing awareness of NEO contamination in WWTPs and numerous studies on its occurrence in WWTP influents and effluents [13,16], several knowledge gaps remain. First, the relative contributions of agricultural, industrial, and domestic sources to NEO loads in urban wastewater are poorly quantified, especially for mNEOs, which are generated through biological or chemical transformations during pesticide use, transport, or treatment. For instance, mNEOs like desnitro-imidacloprid [17] and dinotefuran-urea (DIN-U) [18] may exhibit higher toxicity or persistence than their parent compounds, yet their sources and transformation dynamics in wastewater systems are understudied. Second, although spatial variations in NEOs across WWTPs have been reported [1,19], their relations with regional characteristics (such as land use, population density, and industrial activities) remain uncharacterized, hindering the development of targeted management strategies. To the best of the authors’ knowledge, very limited studies to date have quantified the point-source emissions of NEOs from WWTPs at a regional scale [1]. Therefore, it is necessary to understand their contribution to NEO contamination in receiving water bodies, especially in rapidly urbanizing regions where agricultural, industrial, and domestic activities intersect. However, current emission models for WWTP-derived NEOs often rely on sufficient measured data [1,20], such as comprehensive influent-effluent datasets, and a novel emission estimation and prediction model is needed under data-limited conditions.

This study demonstrates that effluent emissions can be effectively predicted using influent data, plant-scale process information, and literature-based removal efficiencies within a probabilistic Monte Carlo framework. It aims to systematically investigate the occurrence, sources, and transformation of NEOs in WWTPs in suburban Shanghai, an area characterized by diverse land uses and multiple potential NEO exposure pathways. The objectives are to: 1) characterize the spatial distribution of 8 pNEOs and 6 mNEOs in the influents of 21 WWTPs, 2) identify dominant sources and link NEO profiles to agricultural, industrial, and domestic activities, and 3) develop a novel emission model to estimate point-source discharges of NEOs from both sampled and unsampled WWTPs in Shanghai. The developed emission model will be used to couple sampled influent data with regression-based extrapolation to the city scale and incorporate plant-scale process information and literature-based removal efficiencies within a probabilistic Monte Carlo framework. This approach highlights the model applicability when direct discharge data are unavailable, which distinguishes it from conventional single-plant or fixed-factor approaches and represents one of its methodological innovations. The findings offer valuable insights for urbanized regions and cities facing emerging contaminant challenges, with implications for integrated strategies of source control and management, treatment process optimization, and regional pollution mitigation to address the multifaceted threats of NEOs in water cycles.

## 2 Methods

### 2.1 Sampling design

**Fig. 1:**
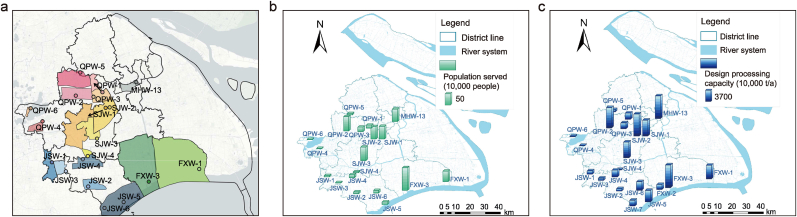
Geographic, demographic, and infrastructural characteristics of the 21 wastewater treatment plants (WWTPs): (a) service areas, (b) served population, and (c) designed treatment capacity. QPW, MHW, SJW, FXW and JSW denote WWTPs in Qingpu, Minhang, Songjiang, Fengxian and Jinshan districts of Shanghai, respectively.

The water samples were collected from 21 major municipal WWTPs in suburban Shanghai, China (Fig. 1), serving a population of approximately 5 million across an area of 1414 km^2^. The land use and land cover (LULC) map of Shanghai can be found in Fig. S1. These facilities have a total designed treatment capacity of 603.35 million t/a, with individual capacities ranging from 912.5 thousand to 73,000 thousand t/a. Sampling was conducted between 4 and 9 October 2022. It should be noted that the early October sampling period was strategically aligned with the post-harvest phase of major autumn crops in Shanghai, specifically designed to capture peak agricultural pesticide inputs following seasonal application cycles. At each sampling site, a comprehensive sampling strategy was employed to ensure representativeness. Influent samples (approximately 300 mL each) were obtained by manually collecting two discrete samples (one in the morning and one in the afternoon) and combining them in equal volumes in order to mitigate the effects of short-term fluctuations in water quality. A total of 112 samples were collected, and the sampling details can be found in Table S1. All water samples were placed in brown glass bottles to protect against photolytic degradation of NEOs. The samples were then stored at −20 °C during transportation and subsequent laboratory storage to minimize biological and chemical activity. All samples were analyzed within one week of arrival at the laboratory.

### 2.2 Extraction, cleanup, and instrumental analysis

The liquid-liquid extraction method was used to extract NEOs from water samples, as described in our previous studies [1,7]. Since no isotope-labeled internal standards of neonicotinoid metabolites were commercially available in China, we used imidacloprid-d_4_ and thiamethoxam-d_3_ for the determination of their corresponding parent compounds and metabolites, and clothianidin-d_3_ was used for the quantification of the remaining neonicotinoids due to their structural similarity to clothianidin. Briefly, water samples were placed in a flask with an extraction solvent containing sodium chloride (NaCl), isotope-labeled standards (imidacloprid-*d4*, thiamethoxam-*d3*, and clothianidin-*d3*, used as internal standards and method recovery surrogates) and dichloromethane (CH_2_Cl_2_), and shaken for 5 min. The organic phase in the flask was then collected, and the above extraction process was repeated thrice. The extract was mixed with anhydrous sodium sulfate (Na_2_SO_4_) for dehydration and transferred to a column for further purification. The eluate was concentrated to less than 1 mL and acetonitrile (CH_3_CN) was added, after which the mixture was condensed to about 1 mL again before being transferred to a brown vial for further analysis.

The concentrations of 8 pNEOs and 6 mNEOs were determined by an ultra-performance liquid chromatography coupled with a triple quadrupole mass spectrometer Xevo TQ-S (UPLC-MS/MS, Waters Corporation, Milford, MA, U.S.). Component separation was achieved using a YMC ODS-AQ column (100 mm × 2.1 mm, 3 μm, YMC, Allentown, PA, U.S.), with an electrospray ionization (ESI) source in positive ion mode under multiple reaction monitoring (MRM) and two transition ions (quantitative and validation) for sample analyses. The 8 pNEOs included acetamiprid (ACE), clothianidin (CLO), dinotefuran (DIN), imidacloprid (IMI), imidaclothiz (IMID), nitenpyram (NIT), thiacloprid (THI) and thiamethoxam (THIA), and 6 mNEOs included N-desmethyl-acetamiprid (N-DM-ACE), desnitro-imidacloprid (DN-IMI), 5-hydroxy-imidacloprid (5-OH-IMI), imidacloprid-urea (IMI-urea), N-desmethyl-thiamethoxam (N-DN-THIA) and 1-methyl-3-(tetrahydro-3-furylmethyl) urea (DIN-U). Their detailed physicochemical properties are listed in Table S2.

### 2.3 Quality assurance and quality control

The analytical standards of NEOs were all purchased from Dr. Ehrenstorfer GmbH (Augsburg, Germany). Three isotope-labeled standards (IMI-*d4*, THIA-*d3*, and CLO-*d3*) were purchased from C/D/N Isotopes Inc. (Quebec, Canada). Dichloromethane and acetonitrile were purchased from Fisher Scientific (Leicestershire, UK). Solvent blank analysis was performed on every 10 water samples to monitor the instrument contamination. No instrument contamination was detected during the laboratory analysis. In addition, a procedural blank sample was included in each batch of water samples, and the results of the procedural blank were subtracted from the determined levels. The method recoveries of IMI-*d4* and THIA-*d3* were 75.1% ± 12.5% (62.4%–121%) and 61.0% ± 10.2% (67.8%–86.2%), respectively. The spiked recoveries of individual NEOs were also quantified with their mean recoveries in the range of 94.5% (DIN) to 116% (THI). The recoveries suggest that the determination of pNEOs and mNEOs was acceptable. In addition, the limit of detection (LOD) and limits of quantification (LOQ) ranged from 0.0015 (THI) to 0.0349 ng/L (DIN) and from 0.0050 (THI) to 0.1166 ng/L (DIN), respectively. More detailed information on the recoveries, LOD, and LOQ is provided in Table S3. Concentrations below the LOD/LOQ were assigned a value of zero, representing conservative, low-bound estimates of the actual concentrations and emissions.

### 2.4 Data analysis

Waters MassLynx software v4.1 (Waters Corporation, U.S.) was used for instrument control and data analysis of 8 pNEOs and 6 mNEOs. Statistical analysis of experimental results was performed by SPSS 22.0 software (IBM, U.S.) and RStudio Desktop (version 2022.12.0 + 353, POSIT Corporation, MA, U.S.), including linear correlation analysis (pearson correlation test), hierarchical cluster analysis (using intergroup linkage and squared Euclidean distance), generalized linear model (GLM), univariate analysis of variance (one-way and two-way ANOVA) test, principal component analysis (PCA), two individual sample comparison analysis (*t*-student test). The significance threshold for all statistical tests was set at *p <* 0.05.

### 2.5 Risk assessment

The risk quotient (RQ) method was applied to evaluate the ecological risks posed by 14 NEOs. The RQ value reflects the potential risk level and was calculated using Eq. 1:

(1) RQ = MEC/PNEC

Where, MEC (ng/L) represents the measured environmental concentration of a specific chemical, and PNEC (ng/L) denotes the predicted no-effect concentration. The PNEC values for the targeted 8 pNEOs and 6 mNEOs are listed in Table S2. An RQ ≤ 0.1 indicates negligible risks, 0.1 ≤ RQ < 1 signifies a low-risk level, 1 ≤ RQ < 10 corresponds to a moderate-risk level, and an RQ ≥ 10 indicates a high-risk scenario.

### 2.6 Removal rates, point source emission modeling, and uncertainties

To evaluate the point source emissions of NEOs from WWTP effluents to the receiving water bodies, a point source emission model was developed and applied for prediction based on multiple parameters, which mainly included estimated volumes of WWTP influents, removal rates (RRs) of NEOs during treatment processes, and the volumes of effluents discharged. RRs of NEOs were estimated using Eqs. (2), (3). According to the Annual Report of Shanghaiʼs Drainage Facilities in 2023 [21], the main processes of all WWTPs were roughly classified as AAO (including SBR and Bardenpho) and oxidation ditch, and the RR of UV process was also included if it was applied as a disinfection process in the target WWTP. The RRs were calculated based on literature data as listed in Table S4.

(2) $${RR}_{i\text{WWTP},j\text{NEOs}}=\text{Average}\left({RR}_{\text{Mid}\;\text{of}\;\text{lit}1},{RR}_{\text{Mid}\;\text{of}\;\text{lit}2},{RR}_{\text{Certain}1},{RR}_{\text{Certain}2}\ldots \right)$$

(3) $${RR}_{i\text{WWTP},\text{pNEOs}}=(\sum_{j=1}^{n}{RR}_{i\text{WWTP},j\text{NEOs}})/n$$

Where, ${RR}_{\text{Mid}\;\text{of}\;\text{lit}X}$ represents the median RR in literature *X* if the range of *RR* was provided for the main process, and ${RR}_{\text{Certain}Y}$ represents the RR provided by literature *Y*. *i* and *j* index WWTPs and pNEOs types, respectively. *n* is the total number of (WWTPs, pNEOs) combinations. Due to the lack of experimentally verified removal efficiencies for most mNEOs, their *RR*s were assumed to be equivalent to those of their corresponding pNEOs (i.e., ${RR}_{i\text{WWTP},\text{pNEOs}}$ = ${\;RR}_{i\text{WWTP},\text{mNEOs}}$), following previous modeling practices for transformation products. This approximation may cause over- or underestimation of mNEO emissions because metabolites often differ in stability, polarity, and biodegradability relative to their parent compounds. To account for this uncertainty, RR variability was incorporated into the Monte Carlo simulations, and its implications are explicitly discussed below.

The annual point source emissions of NEOs from 21 WWTPs, as well as from all WWTPs in Shanghai, were further estimated. The relevant data were found in the Annual Report of Shanghaiʼs Drainage Facilities in 2024 [21]. Firstly, linear regression models were established between observed influent concentrations and explanatory variables (e.g., designed capacity, served population). Outliers were identified through visual inspection and leverage diagnostics, and points exerting disproportionate influence were iteratively removed until a stable fit with R^2^ ≥ 0.95 was achieved. Model residuals were used to compute the standard deviation (σ) and corresponding prediction intervals ($y_{i}\pm t_{\alpha /2,n-2}\cdot \sigma \cdot \sqrt{1+\frac{1}{n}+\frac{\left(x_{i}-\bar{x}\right)}{S}}$), which were subsequently incorporated into the Monte Carlo uncertainty analysis. NEO concentrations and their standard deviations of all WWTPs in Shanghai were estimated by the linear relationships for 21 studied WWTPs, except for PDW-1, for which NEO concentrations were measured in our previous study [1]. Furthermore, total point-source emissions (*E*) from all WWTPs across Shanghai were estimated using Eq. 4, derived from regression-based extrapolations between served population, designed capacity, and measured influent concentrations. While this strong correlation supports internal consistency (R^2^ > 0.95), extrapolation beyond the sampled WWTPs may introduce additional variability associated with regional, operational, and temporal differences and lead to uncertainty in city-wide predictions. Future studies could adopt stratified sampling and develop region-specific regression models incorporating industrial activity and land-use indicators. To address these sources of uncertainty and improve spatial representativeness, Monte Carlo simulations (100,000 iterations) incorporated regression residuals, RR variability, design-flow-rate error, and analytical measurement uncertainty. All input uncertainties and their distributions are summarized in Table S5. This probabilistic framework provides confidence intervals for emission estimates and highlights the relative influence of each uncertainty source.

(4) $$E=\sum_{i}^{m}C_{\text{iWWTP},\text{pNEOs}}^{y∼\alpha \times \text{population}+\beta +\varepsilon }\times \left(1-{RR}_{\text{iWWTP},\text{pNEOs}}\right)\times V_{\text{iWWTP}}+\sum_{i}^{m}C_{\text{iWWTP},\text{mNEOs}}^{y∼\alpha_{0}\times \text{population}+\beta_{0}+\varepsilon_{0}}\times \left(1-{RR}_{\text{iWWTP},\text{mNEOs}}\right)\times V_{\text{iWWTP}}$$

Where, *C* (ng/L) represents the concentrations of NEOs, m represents the number of WWTPs, α and $\alpha_{0}$ represent the coefficients of the linear regression, β and $\beta_{0}$ (ng/L) represent the intercepts of the linear regression, and ε and $\varepsilon_{0}$ represent the residuals of the linear regression. ${RR}_{\text{iWWTP},\text{pNEOs}}$ and ${RR}_{\text{iWWTP},\text{mNEOs}}$ represent the removal rate of pNEOs and mNEOs in WWTP influent and effluent, respectively. *V*_iWWTP_ (×10^7^ L) represents the discharge volume of WWTP influent.

## 3 Results and discussion

### 3.1 Spatial distribution and differences

As detailed in Table S6, considerable concentrations of pNEOs and mNEOs were identified in the influents of 21 WWTPs in suburban Shanghai, with mean concentrations of 568.17 ng/L (range: 170.97–1089.95 ng/L) and 478.20 ng/L (range: 133.87–899.78 ng/L), respectively. All 8 target pNEOs and 6 mNEOs were detected in these WWTPs, with 5 pNEOs (THIA, IMID, IMI, ACE, and DIN) showing ubiquitous presence (100% detection frequency), followed by NIT and THI (95%) as well as CLO (57%). The 100% detection frequency for major pNEOs must be interpreted in light of the high sensitivity of the applied analytical method, with exceedingly low LODs (0.0015–0.0349 ng/L). Although potential biases like background contamination exist, stringent quality controls were applied. Together with the high environmental concentrations measured, the results strongly indicate the extensive usage of NEOs in the suburban environment of Shanghai**.** Among mNEOs, three compounds (IMI-urea, DN-IMI, and N-DM-ACE) were detected across all WWTPs, followed by DIN-U (90%), N-DN-THIA (76%), and 5-OH-IMI (19%). These findings indicate significant biotic and abiotic transformation of NEOs during their transport through domestic sewer systems and urban stormwater runoff prior to WWTP entry, consistent with previous studies [22,23].

**Fig. 2:**
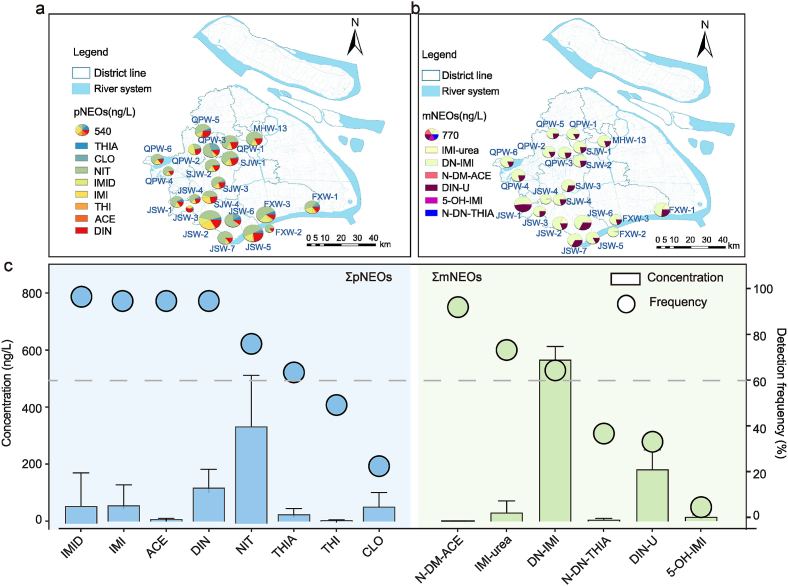
Spatial distribution of (a) ΣpNEOs and (b) ΣmNEOs in influents of 21 WWTPs; (c) detection frequency and average concentrations of NEOs (n = 112).

**Fig. 3:**
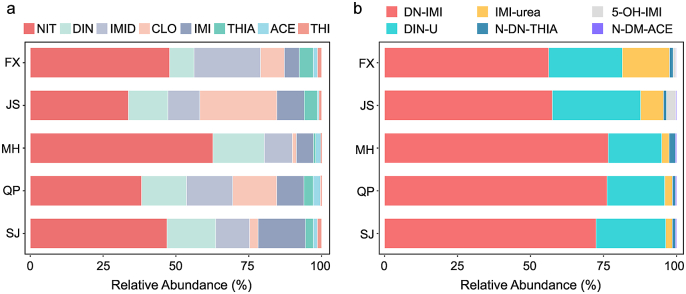
Relative abundance of (a) pNEOs and (b) mNEOs in WWTP influents across five districts.

The spatial distribution of NEOs revealed that the highest total pNEOs (ΣpNEOs) and ΣmNEOs concentrations both fell in JSW-2, located in the JS district, reaching 1089.95 ± 625.74 ng/L and 899.78 ± 108.69 ng/L, respectively (Fig. 2a and b). This is most likely attributed to its highest agricultural acreage (6009.17 hm^2^ [24]) in this district and the presence of pharmaceutical industries, including pesticide industries, as illustrated in Fig. S1. Notably, the JS district established national-level modern agricultural and agroecological parks in 2022, which likely necessitated intensive NEO applications for crop and vegetation protection. These compounds could enter municipal wastewater through both sanitary sewer systems and urban stormwater runoff. Fig. 3 shows a consistent composition profile of pNEOs and mNEOs across districts, with NIT and DIN of the major pNEOs and DN-IMI and DIN-U prevailing among mNEOs. The abundance of NIT and DIN correlates with their higher aqueous solubility (Table S2), suggesting agricultural and industrial sources as major contributors in suburban Shanghai. The absence of N-DM-ACE associated with human urinary excretion [25] as a biomarker of exposure [26], may indicate a low direct contribution from domestic sewage.

As shown in Fig. 2c, individual pNEO concentrations ranged from <LOD to 894.87 ng/L, with NIT exhibiting the highest mean concentration at 380.83 ng/L, accounting for 55.3% of the total pNEOs. In contrast, mNEO concentrations were in the range of <LOD to 971.75 ng/L, with DN-IMI dominating at an average of 567.36 ng/L, nearly 3-fold higher than the second most abundant metabolite DIN-U (182.37 ng/L), both exceeding their pNEOs (i.e., IMI and DIN at 56.45 and 117.89 ng/L, respectively). The elevated mean concentrations of DN-IMI and DIN-U reflected a widespread trend across the sampled WWTPs, which is supported by their high detection frequencies and consistently high levels at most sampling sites. The remaining mNEOs showed much lower levels (1.79–30.01 ng/L), all below their corresponding pNEOs.

Statistical analysis (Table S7) revealed significant positive correlations (*p* < 0.01) of THIA with CLO (*r* = 0.435) and THI with IMID (*r* = 0.578) and DIN (*r* = 0.253). Some pNEOs (i.e., IMI and ACE) were also positively related to their mNEOs, i.e., IMI-urea (*p* < 0.01, *r* = 0.492) and N-DM-ACE (*p* < 0.05, *r* = 0.202), indicative of consistent transformation pathways during wastewater conveyance. The observed significant correlations between specific pNEOs and their mNEOs provide mechanistic evidence that these mNEOs are predominantly formed through in-transit transformation of pNEOs within sewer networks or urban watersheds, rather than originating from independent sources. This indicates dynamic contaminant profiles entering WWTPs, reflecting both direct human discharge and microbial transformation along conveyance pathways. From a toxicological perspective, these relationships are particularly important because certain mNEOs (e.g., DN-IMI) exhibit stronger binding affinity to mammalian nicotinic acetylcholine receptors than their pNEOs, implying increased risks through environmental metabolism [27]. Therefore, risk assessments that focus solely on pNEOs may underestimate overall ecological and human health risks associated with the combined burden of parent and more potent transformation products.

As shown in Table S8, the concentrations of pNEOs (568.17 ng/L) and mNEOs (478.2 ng/L) in influents of 21 WWTPs in Shanghai were at least twice those in typical environmental matrices, such as surface water (8.11–291.06 ng/L for pNEOs and 0.24–11.52 ng/L for mNEOs), tap water (32.05 ng/L for pNEOs and 0.26 ng/L for mNEOs), and soils (80.97 ng/L for pNEOs). This pattern is expected because the concentrations of NEOs in WWTP influent reflect the cumulative input from domestic sewage, agricultural runoff, and urban pesticide applications. In a global context, the mean total concentration of pNEOs in Shanghai WWTP influents (568.17 ng/L) was far higher than those in Spain (34.44 ng/L) and the U.S. (213.10 ng/L), but significantly lower than in Qingdao, China (2220.14 ng/L). This disparity can be attributed to distinct regional usage patterns. The exceptionally high level of NIT (370.83 ng/L), which was rarely detected in other Chinese studies, was a primary driver of elevated total concentrations of pNEOs in Shanghai, potentially linked to its prevalent use in urban pest control (e.g., pet flea treatments) and extensive agricultural and horticultural activities in suburban areas. Furthermore, the dominance of DN-IMI (567.36 ng/L) in Shanghai suggests significant environmental transformation of the widely used IMI before entering WWTPs, a phenomenon that is less reported in other regions. These regional contrasts highlight that the NEO profile in suburban Shanghai is characterized by unique local application practices and possibly different environmental fate processes.

The ecological risks resulting from NEO point-source emissions to the receiving river were assessed using the RQ method, as shown in Table S9. The results show that most pNEOs exhibited low ecological risks (RQ < 1), including ACE (RQ = 0.14), CLO (RQ = 0.92), DIN (RQ = 0.18), and THIA (RQ = 0.19). However, IMI showed a notably higher risk quotient (RQ = 7.31), suggesting moderate ecological risk to sensitive aquatic invertebrates. The elevated RQ for IMI likely reflects its strong binding affinity to insect nicotinic acetylcholine receptors and its persistence in aquatic environments. Due to the lack of experimentally established PNECs for most mNEOs, their ecological risks were not quantitatively evaluated. Overall, these results imply that while most pNEOs pose low risk under current emission conditions, imidacloprid remains a compound of potential concern requiring further monitoring and mitigation at WWTP outlets. As global urban population density increases and insecticide resistance becomes more prevalent [28], urban NEO usage is expected to rise, resulting in increasingly severe ecological risks. This underscores the need for improved wastewater treatment strategies, such as Advanced Oxidation Processes (AOPs), bioaugmentation, adsorption [29,30], targeting these contaminants.

### 3.2 Source identification

**Fig. 4:**
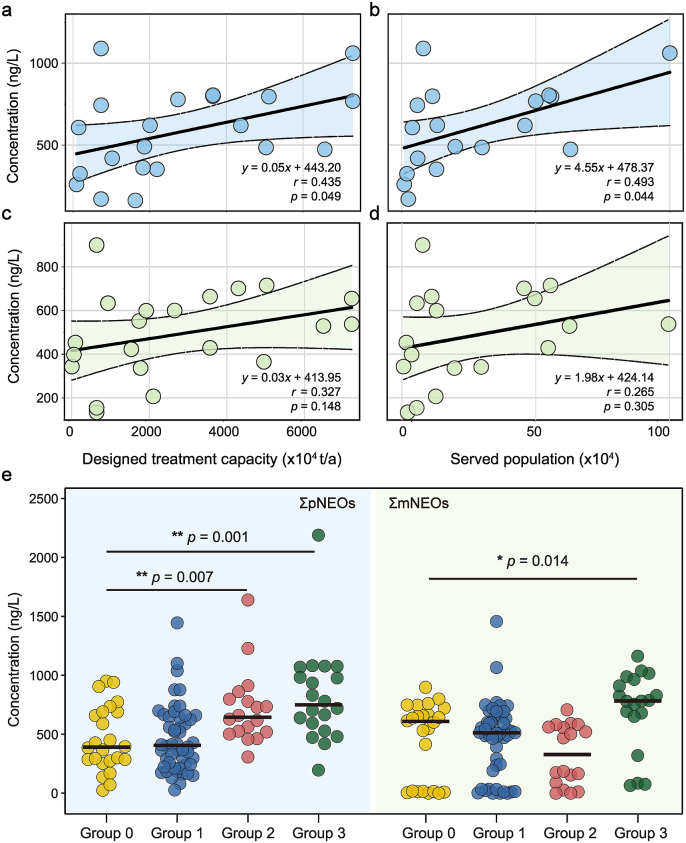
Impacts of designed treatment capacity and served population on the concentrations of (a, b) pNEOs and (c, d) mNEOs in WWTP influents; (e) Concentrations of pNEOs and mNEOs in WWTP influents in different groups. Group 0: no industrial or agricultural source; Group 1: industrial source; Group 2: agricultural source; Group 3: both sources. ∗*p* < 0.05, ∗∗*p* < 0.01.

Generally, there are several potential sources of NEOs in WWTP influents, such as urban runoff [15], household pet-washing wastewater [31], and human urine and feces [32]. Additional potential sources may arise from illegal industrial discharges or illicit connections in separate sewer systems that improperly channel stormwater into municipal wastewater networks [33]. In order to characterize the dominant input sources, the correlations of pNEOs and mNEOs with designed treatment capacity, served population, and service area were analyzed. As shown in Fig. 4, pNEO concentrations exhibited significant positive correlations with both designed treatment capacity (*r* = 0.435, *p* = 0.049) and served population (*r* = 0.493, *p* = 0.044). While mNEO concentrations showed weaker positive trends with these parameters (*r* = 0.327, *p* = 0.148 for capacity; *r* = 0.265, *p* = 0.305 for population), the notable associations for pNEOs strongly suggest contributions from household-scale sources, such as pet washing wastewater and human excretion. These findings align with the hypothesis that residential usage also plays a substantial role in NEO contamination of municipal wastewater, rather than solely agricultural or industrial inputs.

To investigate non-domestic sources of NEOs, WWTPs were classified into four groups based on the land use of their service regions: Group 0 (no industrial/agricultural sources), Group 1 (industrial-dominated), Group 2 (agricultural-dominated), and Group 3 (both industrial and agricultural sources). Two-tailed *t*-tests were performed on the concentrations of pNEOs and mNEOs across these groups. As shown in Fig. 4e, Group 0 exhibited significantly lower pNEO concentrations (461.31 ng/L) compared to Group 2 (718.52 ng/L, *p* = 0.007) and Group 3 (821.72 ng/L, *p* = 0.001), strongly indicating agricultural activities as a primary source of pNEOs. For mNEOs, only Group 0 (446.92 ng/L) and Group 3 (706.62 ng/L) showed significant differences (*p* = 0.014), suggesting that mNEOs arise from combined industrial and agricultural inputs rather than single-source contributions, in addition to biotic transformation processes (e.g., human metabolism in domestic sewage). Industrial sources are likely linked to pesticide manufacturing and formulation processes in sectors such as the chemical and agrochemical industries, particularly in the SJ district. These facilities may discharge NEO-contaminated wastewater directly (legally or illegally) or release pollutants through accidental spills/leaks of raw materials, intermediates, or finished products. The stronger association of mNEOs with industrial sources can be attributed to pesticide production chemistry, such as side reactions during synthesis generating metabolite byproducts rather than parent compounds, while raw material impurities may undergo chemical transformations to form mNEOs. Inadequate removal in industrial wastewater treatment could allow these compounds to persist in municipal influents. Moreover, it is well known that pesticide application in urban greening is a major contributor to NEOs in urban surface water or WWTP influents [34]. The urban green space pesticide use was indirectly evaluated using district-level park green area data in this study due to the unavailability of site-specific green space metrics. Weak positive correlations were observed between pNEOs (*r* = 0.111, *p* = 0.092) and mNEOs (*r* = 0.097, *p* = 0.244) with park green areas (Table S10). However, this finding is tentative due to limited sampling coverage (e.g., only one WWTP was sampled in the MH district). Collectively, agricultural practices, urban greening, and industrial activities, as well as domestic biotic pathways, contribute to NEO contamination in WWTP influents in suburban Shanghai. pNEOs derive predominantly from agricultural sources, whereas mNEOs reflect combined industrial and agricultural inputs, underscoring the need for source-specific mitigation strategies in urban wastewater systems.

It is well known that precipitation also has the potential to introduce pollutants through the sewer network into the WWTP influents due to the wash-off of surface contaminants (e.g., vehicle exhausts and dust on urban roads), pollutants leaching from soils (e.g., agricultural fertilizers and pesticides), as well as the wet deposition of air pollutants [35,36]. Therefore, the NEO concentrations in the case of precipitation and non-precipitation were also analyzed in this study, but no significant differences were observed for either pNEOs or mNEOs (*p* > 0.05). This may be attributed to the minimal precipitation during the sampling period (1–2 mm), which was insufficient to trigger the occurrence of surface runoff or soil leaching. Under these hydrological conditions, pesticide inputs from agricultural and urban green space applications appear to enter WWTPs primarily through sanitary drainage systems rather than stormwater-driven runoff, indicating the critical role of drainage infrastructure in mediating NEO transport within urban water cycles. It should be noted that these findings are inferential, relying on correlation and clustering analyses rather than receptor-based source apportionment methods such as Positive Matrix Factorization (PMF) and Principal Component Analysis-Multiple Linear Regression (PCA-MLR) combined with tracer compounds data, limiting quantitative validation of the inferred contributions. Future investigations will employ receptor modeling approaches and targeted sampling of tracer markers to provide more definitive evidence of mNEO industrial origins.

### 3.3 Transformation mechanisms

**Fig. 5:**
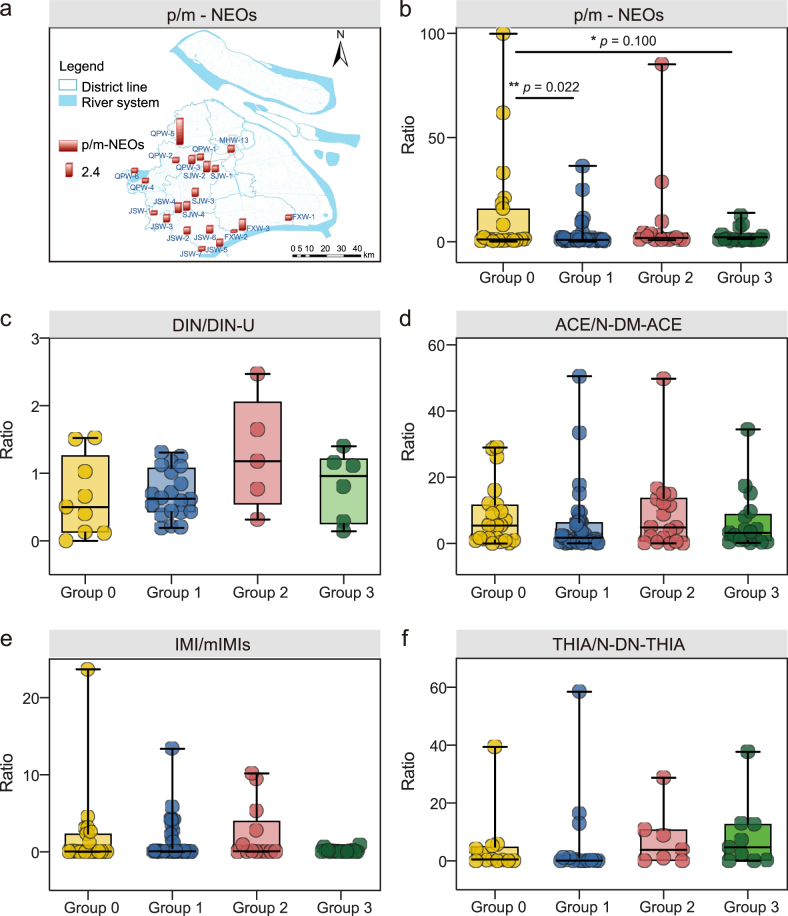
Ratios of pNEOs to their corresponding mNEOs in influents based on (a) individual WWTP and (b) different groups; ratios of individual pNEO to mNEO based on different groups (c-f, mIMIs includes three metabolic derivatives of IMI, i.e., DN-IMI, 5-OH-IMI, and IMI-urea).

To characterize NEO transformation processes, the ratios of pNEO to mNEO (p/m-NEOs) were calculated, and specific compound pairs exhibited distinct degradation profiles (Fig. 5). The DIN/DIN-U and IMI/mIMIs ratios were calculated as 0.65 ± 0.53 and 0.09 ± 3.19, respectively (Fig. 5c,e), indicating that DIN and IMI were well degraded to their metabolic derivatives DIN-U and mIMIs (including DN-IMI, 5-OH-IMI, and IMI-urea), respectively, before entering WWTPs. In contrast, the degradation of THIA and ACE was relatively poor, as reflected by the notably higher THIA/N-DN-THIA and ACE/N-DM-ACE ratios at 4.23 and 4.77, respectively (Fig. 5d,f). Since N-DM-ACE is generally highly abundant in human urine [25], the high ACE/N-DM-ACE ratio may be attributed to the low input of domestic sewage in the studied WWTPs (Fig. 5d). The mean p/m-NEOs ratio in this study was 1.19 (range: 0.39–4.80, Fig. 5a). Values exceeding 1 may be attributed to two primary factors: 1) direct inputs of industrial and agricultural wastewaters containing high-concentration pNEOs without adequate pretreatment, and 2) constrained environmental transformation processes, possibly due to low temperatures during sampling (October) or short hydraulic residence time [37] in drainage pipelines that limit biotic/abiotic degradation. Notably, this ratio is lower than the 2.26 reported for the Yangtze River Delta [1], reflecting regional differences in NEO usage patterns, wastewater management practices, or environmental transformation efficiencies. These findings highlight the complex interplay between source contributions and in-transit degradation processes governing NEO speciation in WWTP influents.

To identify factors influencing p/m-NEOs ratios, a significance analysis was performed after excluding extreme outliers (p/m-NEOs >100). As shown in Fig. 5b, Group 0 (no industrial/agricultural sources) exhibited a significantly higher mean p/m-NEOs ratio (11.82) compared to Group 1 (industrial-dominated, 2.93; *p* = 0.022) and a marginally lower trend relative to Group 3 (combined sources, 2.44; *p* = 0.100), highlighting a stronger influence of industrial inputs on reducing p/m-NEOs ratios. This observation aligns with the hypothesis that industrial processes, such as pesticide synthesis by-reactions and raw material impurities (detailed in Section 3.2), generate substantial mNEOs, coupled with potential pretreatment steps or microbial degradation during pipeline transport that enhance metabolite formation. In contrast, Group 2 (agricultural-dominated) displayed a relatively high mean p/m-NEOs ratio (9.26, *p* = 0.735), indicating elevated pNEO concentrations and limited in-transit metabolism in agricultural wastewater. This is likely due to NEOs from agricultural pesticide applications entering WWTPs primarily through drainage pipelines, where insufficient hydraulic retention time or suboptimal environmental conditions constrain microbial biodegradation, as opposed to agricultural runoff or soil leaching that might facilitate more extensive transformations, under the conditions of this study.

No significant differences were observed across groups in terms of individual pNEO and mNEO pairs (i.e., IMI/mIMIs, DIN/DIN-U, THIA/N-DN-THIA, and ACE/N-DM-ACE), although Group 2 exhibited higher DIN/DIN-U and THIA/N-DN-THIA ratios, indicating that agricultural inputs can influence DIN and THIA metabolism. By contrast, ACE/N-DM-ACE ratios were not affected by source categories, consistent with the fact that N-DM-ACE is primarily derived from human metabolism rather than environmental transformation. It should be noted that the observed ACE/N-DM-ACE ratios (<1 in many cases) are in line with human pharmacokinetics, where ACE is largely excreted unchanged with only partial oxidative N-demethylation to N-DM-ACE [38]. Biomonitoring studies routinely include N-DM-ACE as a biomarker of ACE exposure [39], reinforcing that wastewater-based ACE/N-DM-ACE ratios predominantly reflect human metabolic and excretory profiles rather than in-sewer chemical stability.

### 3.4 Prediction of point source emissions

It has been well-documented that emerging contaminants, including NEOs, are poorly removed by conventional wastewater treatment processes, as indicated in Table S4, where removal efficiencies for different NEOs ranged from 0.8% to 54%. This inefficiency allows these compounds to persist in WWTP effluents, which discharge into urban water bodies as point-source pollutants and contribute to water quality degradation and ecological risks to aquatic life. Despite limited studies, several physical, chemical, and biological techniques have been employed to remove NEOs, with a majority of efforts targeting pNEOs. Among these, chemical oxidation, especially advanced oxidation processes (e.g., electrochemical oxidation and UV-based oxidation), exhibits superior performance, achieving pNEO removal efficiencies of 30%–98% compared to physical processes (e.g., ultrasound, 40%–61%) and biodegradation (0–18%). In contrast, far fewer studies have investigated the removal of mNEOs, with reported efficiencies generally below 50% due to their structural complexity and limited reactivity in conventional treatment systems. Notably, NEO removal efficiency is not solely dependent on treatment technology but also correlates strongly with influent concentrations [16]. High initial contaminant levels can overwhelm treatment capacities, particularly in systems lacking specialized pretreatment for emerging contaminants. These findings underscore the need for integrated strategies that combine source control, improved influent characterization, and targeted advanced treatment technologies to address the dual challenge of pNEO and mNEO contamination—especially mNEOs due to their potentially higher toxicity than pNEOs [40]—in urban water cycles.

**Fig. 6:**
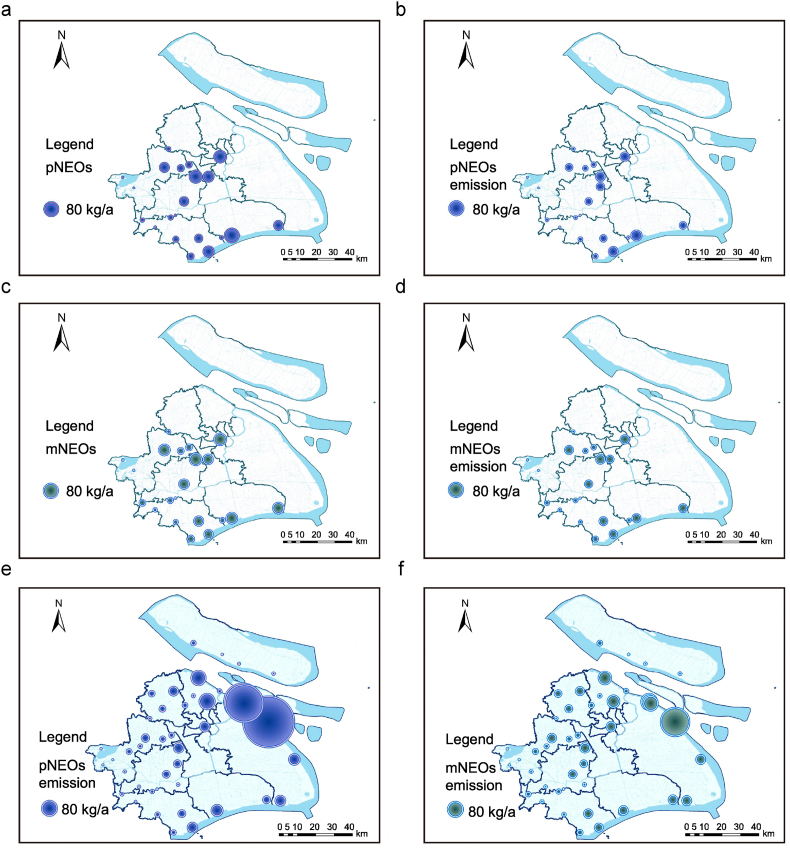
Annual estimates of NEOs in 21 WWTPs: (a) pNEOs in influent, (b) pNEOs in effluent, (c) mNEOs in influent, and (d) mNEOs in effluent; Annual point-source emission estimates of (e) pNEOs and (f) mNEOs from all 21 WWTPs.

To quantify the contribution of NEO point-source discharges to receiving water bodies, annual emissions from 21 WWTPs were estimated using total pollution loads and RRs of NEOs based on previous studies. As presented in Table S11, the RRs of NEOs across these WWTPs were estimated to range from 25% to 48%, indicating variable and relatively low efficiency of conventional treatment processes in mitigating NEO transport. Spatial distribution of estimated annual influent loads of pNEOs and mNEOs, calculated as 465.35 kg/a (range: 0.31–77.87 kg/a) and 475.54 kg/a (range: 0.72–54.78 kg/a), respectively (Fig. 6a–c). Annual emissions of individual pNEO and mNEO from these 21 WWTPs spanned 0.16–42.54 kg and 0.38–29.93 kg, with their total emissions from WWTP effluents estimated at 264.57 kg/a for pNEOs and 269.34 kg/a for mNEOs (Fig. 6b–d). That is, a total of 200.78 kg pNEOs (removal rate: 43.15%) and 206.20 kg mNEOs (removal rate: 43.36%) were removed in WWTP processes per year. These results indicate that traditional WWTP processes partially degrade or remove NEOs, though significant residual concentrations persist in effluents. Notably, these emission estimates rely on measured NEO influent concentrations and estimated RRs based on previous studies, rather than direct effluent measurements. Therefore, a limitation of this study arises from potential in-plant transformation: biodegradation or chemical degradation of pNEOs within WWTPs can generate additional mNEOs, which are not fully captured by models assuming static NEO concentrations. Consequently, mNEO emissions may be underestimated, as treatment-induced metabolite formation could elevate effluent concentrations beyond those inferred from influent data alone.

Across all WWTPs across Shanghai, total annual point-source emissions of pNEOs and mNEOs were estimated at 2947.03 ± 485.62 kg/a and 1056.56 ± 33.37 kg/a, respectively (Table S12). Fig. 6e and f presents their spatial distribution, revealing an obvious disparity: pNEO emissions were nearly 3-fold higher than mNEO emissions. The dominant emission hotspots were concentrated in the PD district, with PDW-1 identified as the largest single source, followed by PDW-5 and PDW-8. This spatial pattern reflects potential regional differences in wastewater composition (e.g., industrial vs. domestic inputs), treatment performance (e.g., processes and removal efficiency variations), and socioeconomic activities (e.g., agricultural intensity, industrial zoning). For example, PDW-1 was identified as the highest emitter, serving the largest population (7.12 million) and exhibiting high influent pNEO concentrations (1790.37 ± 689.32 ng/L). Consequently, its annual emissions reached 953.15 ± 366.42 kg for pNEOs and 294.63 ± 19.15 kg for mNEOs, accounting for 32.3% and 27.9% of the city-wide emissions, respectively (Table S12). In comparison, PDW-2 had similar influent concentrations but a much smaller served population (2.35 million), resulting in considerably lower emissions (385.05 ± 147.58 kg/a of pNEOs). Therefore, the dominance of NEO emissions reflects population/capacity-driven load rather than low removal rates in WWTPs. These findings highlight the need for targeted monitoring and management strategies tailored to high-emission areas, where anthropogenic activities likely drive elevated NEO loads in receiving water bodies.

## 4 Conclusions

This study characterizes the occurrence, sources, and fate of pNEOs and mNEOs in influents of 21 WWTPs in suburban Shanghai. Both pNEOs and mNEOs were detected ubiquitously in all sampled WWTPs. A key finding is that pNEOs were strongly associated with agricultural activities, likely from pesticide applications in farmlands and urban green spaces, while mNEOs reflected a combined influence of industrial processes (e.g., pesticide production by-products) and domestic inputs (e.g., human metabolism) in these WWTPs. Spatial analysis revealed distinct hotspots, with the highest concentrations occurring in areas characterized by intensive agricultural land use and pesticide-related industries. A novel emission model was developed to estimate annual point-source emissions, and the results demonstrated continuous point-source discharges of both pNEOs and mNEOs into receiving water bodies across Shanghai, with pNEOs dominating total loads (total Shanghai-wide emissions reaching 2947.03 kg/a for pNEOs and 1056.56 kg/a for mNEOs). The findings of this study emphasize the need for integrated strategies that combine source control (e.g., optimized pesticide management in agriculture and industry), improved wastewater treatment technologies (e.g., AOPs, bioaugmentation) targeting both pNEOs and mNEOs, and enhanced regional monitoring (particularly in high-emission zones) to mitigate ecological risks associated with these emerging contaminants.

Despite these valuable conclusions and insights, several limitations should be acknowledged. Firstly, sampling was constrained by the COVID-19 epidemic, limiting both temporal coverage and spatial scope. Not all municipal WWTPs in the target region were included, and the sampling period was relatively short, despite being in early October, which is the post-harvest phase of major autumn crops in Shanghai, designed to capture peak agricultural pesticide inputs following seasonal application cycles. Sampling under both wet and dry weather conditions was insufficient to fully characterize runoff-driven NEO inputs. Future studies should investigate seasonal variations and hydrological impacts on contaminant loads. Another limitation arises from potential biotransformation processes of NEOs within treatment systems: pNEOs can undergo biodegradation or chemical degradation during wastewater treatment, generating additional mNEOs. However, concurrent effluent and in-plant sampling data were unavailable due to restricted sampling access, and such transformation cannot be fully captured by influent-based modeling. This oversight highlights the need for future studies to incorporate simultaneous influent, effluent, and in-plant sampling of both pNEOs and mNEOs to accurately quantify transformation dynamics and emission profiles. Such efforts would substantially improve data availability on the degradation of NEOs during WWTP processes, enabling accurate assessment of source emissions of NEOs in the future. Additionally, future studies are recommended to investigate the fate and transport of NEOs across entire urban water systems, including source waters, wastewater networks, and receiving water bodies, to enable more robust assessments of their behavior within urban water cycles.

## CRediT authorship contribution statement

**Yunhui Zhang:** Writing – original draft, Methodology, Funding acquisition, Formal analysis, Data curation, Conceptualization. **Lite Meng:** Visualization, Investigation, Data curation. **Wenfei Yu:** Investigation, Data curation. **Yang Wen:** Writing – review & editing, Visualization, Data curation. **Hui Wang:** Investigation. **Mengchen Sun:** Writing – review & editing, Methodology. **Yuanchen Chen:** Writing – review & editing, Supervision, Methodology, Funding acquisition, Conceptualization. **Bin Dong:** Writing – review & editing, Supervision, Methodology, Funding acquisition, Conceptualization. **Jörg Rinklebe:** Writing – review & editing, Supervision, Conceptualization.

## Conflict of competing interest

The authors declare that they have no known competing financial interests or personal relationships that could have appeared to influence the work reported in this paper.
