# Supplementary material for: Fate, occurrence, and regional-scale emissions of neonicotinoid pesticides and their metabolites in wastewater treatment plants in suburban Shanghai, China
Source: Eco Environ Health. 2026 Jan 20;5(1):100215. doi: 10.1016/j.eehl.2026.100215 (PMC12907088; doi:10.1016/j.eehl.2026.100215)
Supplement: Multimedia component 1 [file mmc1.docx]

**Supplementary Material**

**Fate, distribution, and regional-scale emissions of neonicotinoid pesticides and their metabolites in wastewater treatment plants in suburban Shanghai, China**

**Contents**

**Figure S1** Land use and land cover (LULC) map of Shanghai (Data source: Data Center for Resources and Environmental Sciences, Chinese Academy of Sciences (RESDC, http://www.resdc.cn), 2020)

**Table S1** Details of 21 WWTPs (Ministry of Ecology and Environment of the People’s Republic of China, 2020; Shanghai Municipal Bureau of Water Resources, 2022), sampling information and NEO levels in WWTP influents

**Table S2** Physical and chemical properties of eight individual neonicotinoids and 6 NEO metabolites

**Table S3** The average limits of detection (LOD), limits of quantification (LOQ), method recoveries, and spiked recoveries (%) of different individual neonicotinoids

**Table S4** Removal rates of NEOs by different WWTP treatment processes

**Table S5** Summary of stochastic input parameters and assumed probability distributions used in Monte Carlo simulation

**Table S6** NEO levels in WWTP influents

**Table S7** Correlation among different pNEOs and mNEOs in WWTP influents (n = 112)

**Table S8** Influent concentrations of NEOs and their metabolites in 21 WWTPs in Shanghai compared with other scenarios (ng/L)

**Table S9** Ecological risks resulted from NEO point-source emissions to receiving river

**Table S10** Park green areas and NEO levels in WWTP influents in 5 districts (Shanghai Municipal Bureau of Statistics, 2022)

**Table S11** Annual emission estimates of NEOs from 21 WWTPs to receiving water bodies in suburban Shanghai

**Table S12** Annual emission estimates of NEOs from all other WWTPs to receiving water bodies in Shanghai

**
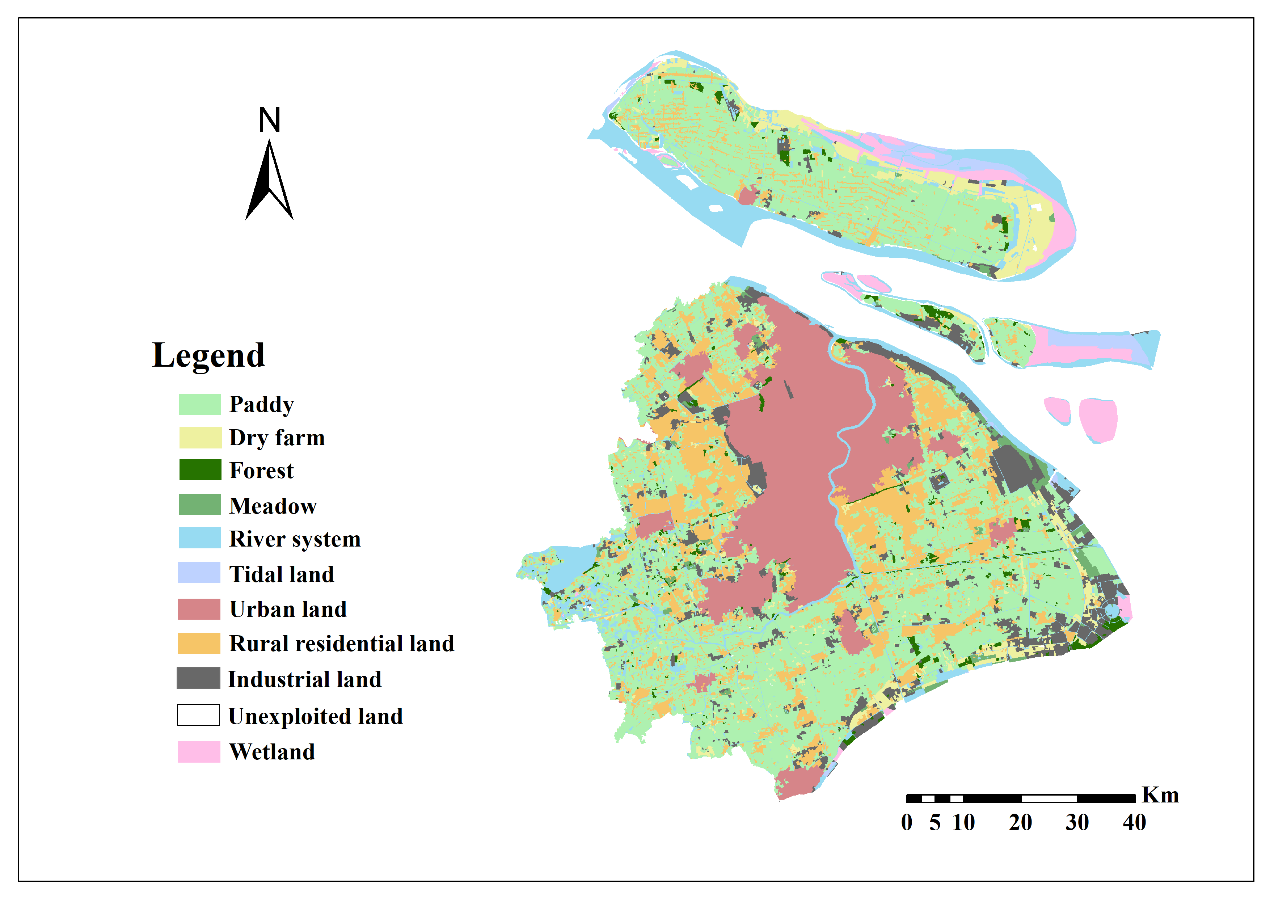
**

**Figure S1** Land use and land cover (LULC) map of Shanghai (Data source: Data Center for Resources and Environmental Sciences, Chinese Academy of Sciences (RESDC, http://www.resdc.cn), 2020)

**Table S1** Details of 21 WWTPs (Ministry of Ecology and Environment of the People’s Republic of China, 2020; Shanghai Municipal Bureau of Water Resources, 2022), sampling information and NEO levels in WWTP influents

| **No.** | **WWTP** | **District** | **Longitude (°E)** | **Latitude (°N)** | **Capacity (×10^4^ t/y)** | **Served population (×10^4^)** | **Serving area**  **(km^2^)** | **Treatment processes** | **Sampling date** | **Sampling size** |
| --- | --- | --- | --- | --- | --- | --- | --- | --- | --- | --- |
| 1 | FXW-1 | Fengxian | 121.74 | 30.87 | 4380 | 46 | 313 | AAO+advanced treatment | 5−9 Oct | 5 |
| 2 | FXW-2 |  | 121.48 | 30.81 | 1624.3 |  |  | Biochemical treatment+activated carbon adsorption+AOP |  | 5 |
| 3 | FXW-3 |  | 121.5 | 30.82 | 7300 | 100 | 289 | AO+filtration | 5, 7, 8 Oct | 3 |
| 4 | JSW-1 | Jinshan | 121.03 | 30.90 | 1022 | 6 | 12.63 | AAO+Aeration | 5−9 Oct | 5 |
| 5 | JSW-2 |  | 121.20 | 30.80 | 730 | 8.1 | 20.3 | Hydrolysis acidification+UCTMBR+chlorination |  | 5 |
| 6 | JSW-3 |  | 121.10 | 30.86 | 730 | 2.5 | 10.48 | Oxidation ditch |  | 5 |
| 7 | JSW-4 |  | 121.18 | 30.91 | 2190 | 13 |  | Modified oxidation ditch+circulating clarifier+cloth filtration |  | 5 |
| 8 | JSW-5 |  | 121.38 | 30.74 | 3650 | 11.5 | 155 | Modified SBR |  | 5 |
| 9 | JSW-6 |  | 121.32 | 30.80 | 2737.5 |  |  |  |  | 5 |
| 10 | JSW-7 |  | 121.28 | 30.71 | 1825 |  |  | Oxidation ditch; AAO+MBR |  | 5 |
| 11 | MHW-13 | Minhang | 121.44 | 31.23 | 7300 | 50-55 | 67 | Modified AAO+advanced treatment | 4−9 Oct | 6 |
| 12 | QPW-1 | Qingpu | 121.27 | 31.19 | 2007.5 | 13.3 | 38.16 | AAO |  | 6 |
| 13 | QPW-2 |  | 121.15 | 31.18 | 6570 | 63 | 93.2 | Primary sedimentation-secondary sedimentation- magnetic coagulation-denitrification filtration |  | 6 |
| 14 | QPW-3 |  | 121.23 | 31.17 | 1861.5 | 20 | 47.64 | AAO+advanced treatment |  | 6 |
| 15 | QPW-4 |  | 120.99 | 31.07 | 91.25 | 1 | 51.71 | MSBR |  | 6 |
| 16 | QPW-5 |  | 121.17 | 31.27 | 730 | 5.9 | 57.63 | AAO |  | 6 |
| 17 | QPW-6 |  | 120.93 | 31.12 | 182.5 | 2 |  | MSBR |  | 6 |
| 18 | SJW-1 | Songjiang | 121.31 | 31.13 | 5110 | 55.9 |  | Reverse AAO | 4, 5, 7, 8 Oct | 4 |
| 19 | SJW-2 |  | 121.10 | 31.00 | 3650 | 55 | 193.6 | Reverse AAO | 4−9 Oct | 6 |
| 20 | SJW-3 |  | 121.25 | 31.00 | 5037 | 30 | 60 | AO |  | 6 |
| 21 | SJW-4 |  | 121.2 | 30.93 | 146 | 4 | 4.47 | AAO |  | 6 |

**Table S2** Physical and chemical properties of 8 individual neonicotinoids and 6 NEO metabolites

| **Compounds** | **Chemical Structure** | **Molecular Mass**  **(g/mol)** | **Solubility (g/L)** | **log *K*_ow_** | **PNEC (ng/L)** |
| --- | --- | --- | --- | --- | --- |
| Acetamiprid (ACE) | 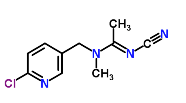 | 222.7 | 4.2 | 0.8 | 62 |
| Clothianidin (CLO) | 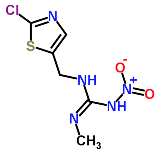 | 249.7 | 0.327 | 0.7 | 56 |
| Dinotefuran (DIN) | 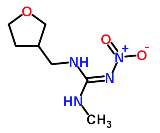 | 202.2 | 54.3 | -0.64 | 658 |
| Imidacloprid (IMI) | 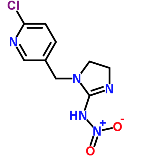 | 255.7 | 0.51 | 0.57 | 7.72 |
| Imidaclothiz (IMID) | 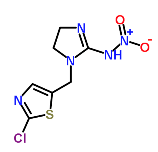 | 261.7 | 5 | NA | NA |
| Nitenpyram (NIT) | 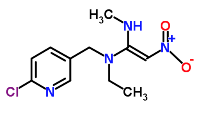 | 270.7 | 840 | -0.64 | NA |
| Thiacloprid (THI) | 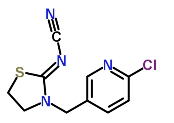 | 252.7 | 0.185 | 1.26 | 38 |
| Thiamethoxam (THIA) | 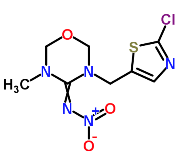 | 291 | 4.1 | -0.13 | 136 |
| N-desmethyl-acetamiprid (N-DM-ACE) | 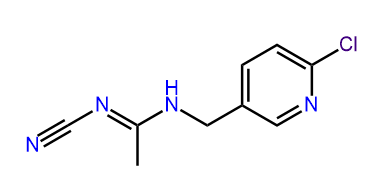 | 208.7 | 0.3998 | 2.34 | NA |
| Desnitro-imidacloprid (DN-IMI) | 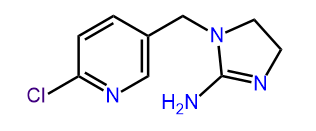 | 210.7 | 3.952 | -0.6 | NA |
| 5-hydroxy imidacloprid (5-OH-IMI) | 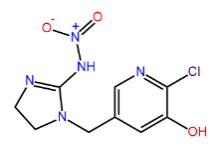 | 271.7 | 183.25 | -0.89 | NA |
| Imidacloprid-urea (IMI-urea) | 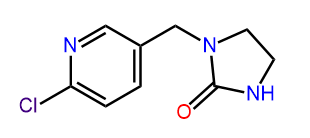 | 211.7 | 47.892 | 0.64 | NA |
| N-desmethyl-thiamethoxam (N-DN-THIA) | 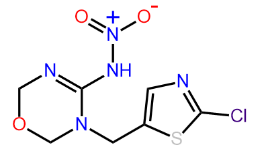 | 277.7 | 1000 | -0.93 | NA |
| 1-methyl-3-(tetrahydro-3-furylmethyl) urea (DIN-U) | 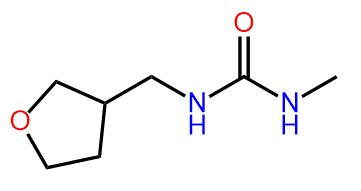 | 158.2 | 1000 | -0.1 | NA |

NA: not available.

**Table S3** The average limits of detection (LOD), limits of quantification (LOQ), method recoveries, and spiked recoveries (%) of different individual neonicotinoids

| **Compounds** | **Calibration curve** | **R^2^** | **Weighted calibration R^2^** | **Recovery (SD, %)** | **LOD (ng/L)** | **LOQ (ng/L)** |
| --- | --- | --- | --- | --- | --- | --- |
| ACE | y = 23.111x – 0.0318 | 0.9997 | 0.9996 | 116 (4.5) | 0.0017 | 0.0058 |
| IMI | y = 4.4391x – 0.0032 | 0.9996 | 0.9995 | 101.0 (2.0) | 0.0062 | 0.0208 |
| IMID | y = 4.7361x +0.0333 | 0.9994 | 0.9993 | 114 (3.6) | 0.0063 | 0.0210 |
| CLO | y = 2.6683x + 0.0117 | 0.9996 | 0.9995 | 98.0 (2.4) | 0.0123 | 0.0412 |
| THI | y = 24.109x – 0.0169 | 0.9996 | 0.9995 | 116 (1.6) | 0.0015 | 0.0050 |
| NIT | y = 11.506x – 0.1264 | 0.9976 | 0.9972 | 104 (11.5) | 0.0200 | 0.0668 |
| THIA | y = 4.2898x – 0.0162 | 0.9994 | 0.9992 | 96.8 (1.0) | 0.0074 | 0.0249 |
| DIN | y = 5.5046x – 0.0681 | 0.9964 | 0.9957 | 94.5 (6.3) | 0.0349 | 0.1166 |
| IMI-*d4* | - | - | - | 75.1 (12.5) | - | - |
| THIA-*d3* | - | - | - | 61 (10.2) | - | - |

Note: The means and standard deviations (SD) of the spiked recoveries were calculated from 10 blanks with four concentration levels (0, 0.5, 5, and 50 ng/L). The eight individual neonicotinoids are acetamiprid (ACE), thiamethoxam (THIA), imidacloprid (IMI), clothianidin (CLO), thiacloprid (THI), dinotefuran (DIN), nitenpyram (NIT), and imidaclothiz (IMID). IMI-*d4* and THIA-*d3* are isotope-labeled standards. Method recoveries were calculated from IMI-*d4* and THIA-*d3*. Spiked recoveries were calculated from the eight standard analytes.

**Table S4** Removal rates of NEOs by different WWTP treatment processes

| **NEO** | **Processes** | **Removal rate** | **Ref.** |
| --- | --- | --- | --- |
| ACE | Ultrasound, pH = 7.79, t = 120 min | 50% | (Domínguez et al., 2021b) |
|  | Electrochemical oxidation, 20 ℃, t = 60 min | 48%−85% | (Domínguez et al., 2021a) |
|  | Only UV, UV+PDS or H_2_O_2_, natural pH, 20 °C | 70%−82% | (Acero et al., 2019) |
|  | Primary sedimentation-aeration-secondary sedimentation-chlorine disinfection | 51%−54% | (Sadaria et al., 2016) |
|  | Primary sedimentation-aeration/FeCl_3_ oxidation- secondary sedimentation | 23.2% | (Iancu and Radu, 2018) |
|  | Aeration-coagulation-flocculation-precipitation-sand filtration-chlorination in drinking water treatment plant | 40.1% | (Wan et al., 2019) |
|  | Municipal: Grille, AAO, sedimentation, UV photolysis/chlorination | 0% | (Qu et al., 2024) |
|  | Municipal: Grille, AAO/oxidation ditch, sedimentation, (biofilter/filter), chlorination/UV photolysis, (artificial wetland) | 32.2% | (Li et al., 2022) |
| CLO | Biodegradation, aerobic conditions, 5 g/L biomass, 25 °C, pH = 7.5 | 0−18% | (Gusmaroli et al., 2020) |
|  | Primary sedimentation-aeration-secondary sedimentation-chlorine disinfection | 22%−38% | (Sadaria et al., 2016) |
|  | Aeration-coagulation-flocculation-precipitation-sand filtration-chlorination in drinking water treatment plant | 4.3% | (Wan et al., 2019) |
|  | Municipal: Grille, AAO, sedimentation, UV photolysis/chlorination | 100% | (Qu et al., 2024) |
|  | Municipal: Grille, AAO/oxidation ditch, sedimentation, (biofilter/filter), chlorination/UV photolysis, (artificial wetland) | 81.5% | (Li et al., 2022) |
| DIN | Primary sedimentation-aeration/FeCl_3_ oxidation- secondary sedimentation | 23.2% | (Iancu and Radu, 2018) |
|  | Municipal: Grille, AAO, sedimentation, UV photolysis/chlorination | 73% | (Qu et al., 2024) |
|  | Municipal: Grille, AAO/oxidation ditch, sedimentation, (biofilter/filter), chlorination/UV photolysis, (artificial wetland) | 49.5% | (Li et al., 2022) |
| IMI | Ultrasound, pH = 7.79, t = 120 min | 55% | (Domínguez et al., 2021b) |
|  | Electrochemical oxidation, 20 ℃, t = 60 min | 85%−96% | (Domínguez et al., 2021a) |
|  | Primary sedimentation-aeration/FeCl_3_ oxidation- secondary sedimentation | 22.4% | (Iancu and Radu, 2018) |
|  | Primary sedimentation-aeration-secondary sedimentation-chlorine disinfection | 12% | (Sadaria et al., 2016) |
|  | Ozonation-activated carbon | 30% | (Wan et al., 2020) |
|  | Aeration-coagulation-flocculation-precipitation-sand filtration-chlorination in drinking water treatment plant | 0.8% | (Wan et al., 2020) |
|  | Municipal: Grille, AAO, sedimentation, UV photolysis/chlorination | 26% | (Qu et al., 2024) |
|  | Industrial: Grille, (regulating), (hydrolysis acidification), (AAO/A-O), sedimentation, (Fenton), (biofilter), (O_3_), (BAF), (chlorination/UV photolysis) | 86.9% | (Li et al., 2022) |
|  | Municipal: Grille, AAO/oxidation ditch, sedimentation, (biofilter/filter), chlorination/UV photolysis, (artificial wetland) | 34.6% |  |
| IMID | Primary sedimentation-aeration-secondary sedimentation-chlorine disinfection | 12%−37% | (Sadaria et al., 2016) |
|  | Industrial: Grille, hydrolysis acidification, AAO, sedimentation, UV photolysis/chlorination | 85% | (Qu et al., 2024) |
|  | Municipal: Grille, AAO, sedimentation, UV photolysis/chlorination | 20% |  |
|  | Industrial: Grille, (regulating), (hydrolysis acidification), (AAO/A-O), sedimentation, (Fenton), (biofilter), (O_3_), (BAF), (chlorination/UV photolysis) | 97.1% | (Li et al., 2022) |
|  | Municipal: Grille, AAO/oxidation ditch, sedimentation, (biofilter/filter), chlorination/UV photolysis, (artificial wetland) | 97% |  |
| THI | Ultrasound, pH = 7.79, t = 120 min | 61% | (Domínguez et al., 2021b) |
|  | Electrochemical oxidation, 20 ℃, t = 60 min | 97%−98% | (Domínguez et al., 2021a) |
|  | Biodegradation, aerobic conditions, 5 g/L biomass, 12 °C, pH = 7.5 | 3.8%−13.2% | (Gusmaroli et al., 2020) |
|  | Aeration-coagulation-flocculation-precipitation-sand filtration-chlorination in drinking water treatment plant | 20% | (Wan et al., 2019) |
|  | Industrial: Grille, hydrolysis acidification, AAO, sedimentation, UV photolysis/chlorination | 31% | (Qu et al., 2024) |
|  | Municipal: Grille, AAO, sedimentation, UV photolysis/chlorination | 68% |  |
| THIA | Ultrasound, pH = 7.79, t = 120 min | 40% | (Domínguez et al., 2021b) |
|  | Electrochemical oxidation, 20 ℃, t = 60 min | 75%−95% | (Domínguez et al., 2021a) |
|  | UV, natural pH, 20 ℃ | 90%−95% | (Acero et al., 2019) |
|  | Primary sedimentation-aeration/FeCl_3_ oxidation- secondary sedimentation | 20.3% | (Iancu and Radu, 2018) |
|  | Industrial: Grille, hydrolysis acidification, AAO, sedimentation, UV photolysis/chlorination | 100% | (Qu et al., 2024) |
|  | Municipal: Grille, AAO, sedimentation, UV photolysis/chlorination | 55% |  |
|  | Industrial: Grille, (regulating), (hydrolysis acidification), (AAO/A-O), sedimentation, (Fenton), (biofilter), (O_3_), (BAF), (chlorination/UV photolysis) | 95.6% | (Li et al., 2022) |
|  | Municipal: Grille, AAO/oxidation ditch, sedimentation, (biofilter/filter), chlorination/UV photolysis, (artificial wetland) | 51.1% |  |
| NIT | Industrial: Grille, hydrolysis acidification, AAO, sedimentation, UV photolysis/chlorination | 75% | (Qu et al., 2024) |
|  | Municipal: Grille, AAO, sedimentation, UV photolysis/chlorination | 100% |  |
|  | Industrial: Grille, (regulating), (hydrolysis acidification), (AAO/A-O), sedimentation, (Fenton), (biofilter), (O_3_), (BAF), (chlorination/UV photolysis) | 86.4% | (Li et al., 2022) |
|  | Municipal: Grille, AAO/oxidation ditch, sedimentation, (biofilter/filter), chlorination/UV photolysis, (artificial wetland) | 80.4% |  |
| N-DM-ACE | Aeration-coagulation-flocculation-precipitation-sand filtration-chlorination in drinking water treatment plant | 3.6% | (Wan et al., 2019) |
| DN-IMI | Ozonation-activated carbon | 25%−50% | (Wan et al., 2019) |

**Table S5** Summary of stochastic input parameters and assumed probability distributions used in Monte Carlo simulation

| **Parameter** | **Probability Distribution** | **Distribution Parameters** |
| --- | --- | --- |
| NEO concentration | Normal | Mean = Predicted value  SD = Standard error of the regression |
| Removal rate | Beta | α and β, derived from the mean (μ) and variance (σ²) of literature data for each specific pollutant-treatment process combination |
| Discharge volume | Triangular | Min = Report value×0.9  Mode = Report value  Min = Report value×1.1 |

**Table S6** NEO levels in WWTP influents

| **No.** | **WWTP** | **District** | **pNEOs (ng/L)** | | | **mNEOs (ng/L)** | | |
| --- | --- | --- | --- | --- | --- | --- | --- | --- |
|  |  |  | **Median** | **AM±STD** | **GM** | **Median** | **AM±STD** | **GM** |
| 1 | FXW-1 | Fengxian | 528.95 | 619.67±266.14 | 583.51 | 826.19 | 701.52±361.08 | 536.67 |
| 2 | FXW-2 |  | 162.00 | 162.85±112.90 | 120.73 | 496.46 | 422.65±218.64 | 302.42 |
| 3 | FXW-3 |  | 1039.45 | 1061.24±373.39 | 1016.48 | 527.50 | 537.69±48.24 | 536.27 |
| 4 | JSW-1 | Jinshan | 415.60 | 419.88±91.91 | 411.84 | 492.68 | 633.20±533.75 | 184.23 |
| 5 | JSW-2 |  | 832.68 | 1089.95±625.74 | 985.40 | 897.41 | 899.78±108.69 | 894.85 |
| 6 | JSW-3 |  | 176.18 | 170.97±31.58 | 168.61 | 22.87 | 133.87±261.21 | 22.23 |
| 7 | JSW-4 |  | 364.64 | 352.63±37.83 | 350.90 | 11.75 | 207.05±276.33 | 26.13 |
| 8 | JSW-5 |  | 935.41 | 798.20±369.40 | 684.61 | 755.98 | 664.02±196.64 | 631.78 |
| 9 | JSW-6 |  | 720.48 | 779.06±224.03 | 747.55 | 652.73 | 599.81±517.35 | 332.57 |
| 10 | JSW-7 |  | 212.78 | 362.30±299.05 | 292.32 | 393.65 | 551.82±354.62 | 467.72 |
| 11 | MHW-13 | Minhang | 733.32 | 768.34±150.45 | 756.24 | 661.89 | 654.71±102.29 | 648.01 |
| 12 | QPW-1 | Qingpu | 624.34 | 621.33±157.67 | 604.85 | 619.17 | 599.10±86.30 | 593.71 |
| 13 | QPW-2 |  | 479.15 | 473.94±219.05 | 429.47 | 612.47 | 528.86±253.15 | 385.81 |
| 14 | QPW-3 |  | 439.61 | 491.02±186.21 | 461.12 | 307.49 | 336.45±365.01 | 49.48 |
| 15 | QPW-4 |  | 279.72 | 260.72±94.34 | 245.20 | 316.22 | 343.23±364.41 | 93.93 |
| 16 | QPW-5 |  | 754.42 | 743.64±142.24 | 731.42 | 121.95 | 154.77±168.38 | 83.19 |
| 17 | QPW-6 |  | 291.08 | 325.16±314.58 | 192.81 | 526.53 | 453.30±378.33 | 97.50 |
| 18 | SJW-1 | Songjiang | 716.74 | 795.69±207.98 | 777.78 | 706.98 | 714.79±26.56 | 714.43 |
| 19 | SJW-2 |  | 681.65 | 804.86±425.85 | 735.39 | 552.27 | 428.86±278.77 | 180.66 |
| 20 | SJW-3 |  | 529.60 | 485.79±151.39 | 460.41 | 355.20 | 341.86±226.82 | 216.49 |
| 21 | SJW-4 |  | 514.67 | 607.08±321.32 | 552.19 | 530.23 | 398.73±245.05 | 159.91 |
| 21 WWTPs | | Shanghai | 528.20 | 568.17±345.40 | 459.79 | 543.78 | 478.20±325.08 | 210.31 |

Note: AM-arithmetic mean; STD-standard deviation; GM-geometric mean.

**Table S****7** Correlation among different pNEOs and mNEOs in WWTP influents (n = 112)

|  | **pNEOs** | | | | | | | | **mNEOs** | | | | | |
| --- | --- | --- | --- | --- | --- | --- | --- | --- | --- | --- | --- | --- | --- | --- |
|  |  | **CLO** | **NIT** | **IMID** | **IMI** | **THI** | **ACE** | **DIN** | **IMI-urea** | **DN-IMI** | **N-DM-ACE** | **DIN-U** | **5-OH-IMI** | **N-DN-THIA** |
| **THIA** | r | 0.435 | 0.238 | -0.064 | 0.032 | -0.04 | -0.02 | -0.015 | 0.069 | 0.133 | 0.007 | -0.022 | 0.463 | 0.02 |
|  | Sig. | 0.000** | 0.012* | 0.504 | 0.735 | 0.673 | 0.837 | 0.872 | 0.467 | 0.161 | 0.945 | 0.816 | 0.000** | 0.835 |
| **CLO** | r |  | 0.087 | -0.1 | -0.065 | -0.07 | 0.167 | -0.08 | 0.229 | 0.209 | 0.01 | 0.072 | 0.34 | 0.049 |
|  | Sig. |  | 0.361 | 0.296 | 0.497 | 0.465 | 0.079 | 0.402 | 0.015* | 0.027* | 0.913 | 0.451 | 0.000** | 0.608 |
| **NIT** | r |  |  | 0.057 | 0.189 | 0.196 | -0.009 | 0.105 | 0.092 | 0.227 | 0.143 | 0.091 | 0.172 | 0.222 |
|  | Sig. |  |  | 0.551 | 0.046* | 0.038* | 0.926 | 0.272 | 0.337 | 0.016* | 0.133 | 0.337 | 0.07 | 0.019* |
| **IMID** | r |  |  |  | 0.033 | 0.578 | -0.136 | 0.18 | 0.21 | -0.026 | -0.059 | 0.041 | -0.08 | 0.196 |
|  | Sig. |  |  |  | 0.73 | 0.000** | 0.153 | 0.057 | 0.026* | 0.789 | 0.538 | 0.67 | 0.404 | 0.038* |
| **IMI** | r |  |  |  |  | 0.009 | 0.109 | 0.175 | **0.492** | 0.049 | -0.015 | 0.139 | -0.04 | 0.022 |
|  | Sig. |  |  |  |  | 0.922 | 0.252 | 0.064 | **0.000**** | 0.609 | 0.876 | 0.144 | 0.675 | 0.818 |
| **THI** | r |  |  |  |  |  | -0.07 | 0.253 | 0.057 | -0.073 | 0.089 | -0.039 | -0.048 | 0.091 |
|  | Sig. |  |  |  |  |  | 0.466 | 0.007** | 0.549 | 0.443 | 0.349 | 0.68 | 0.612 | 0.34 |
| **ACE** | r |  |  |  |  |  |  | 0.122 | 0.184 | -0.246 | **0.202** | -0.065 | -0.088 | 0.047 |
|  | Sig. |  |  |  |  |  |  | 0.201 | 0.053 | 0.009** | **0.032*** | 0.499 | 0.357 | 0.62 |
| **DIN** | r |  |  |  |  |  |  |  | 0.053 | -0.117 | 0.177 | -0.015 | -0.08 | 0.102 |
|  | Sig. |  |  |  |  |  |  |  | 0.582 | 0.218 | 0.061 | 0.879 | 0.401 | 0.286 |
| **IMI-urea** | r |  |  |  |  |  |  |  |  | 0.209 | -0.087 | 0.001 | 0.008 | 0.214 |
|  | Sig. |  |  |  |  |  |  |  |  | 0.027* | 0.36 | 0.994 | 0.934 | 0.023* |
| **DN-IMI** | r |  |  |  |  |  |  |  |  |  | -0.088 | 0.009 | 0.109 | 0.078 |
|  | Sig. |  |  |  |  |  |  |  |  |  | 0.355 | 0.926 | 0.253 | 0.415 |
| **N-DM-ACE** | r |  |  |  |  |  |  |  |  |  |  | 0.255 | -0.009 | -0.094 |
|  | Sig. |  |  |  |  |  |  |  |  |  |  | 0.007** | 0.924 | 0.327 |
| **DIN-U** | r |  |  |  |  |  |  |  |  |  |  |  | -0.003 | -0.024 |
|  | Sig. |  |  |  |  |  |  |  |  |  |  |  | 0.974 | 0.801 |
| **5-OH-IMI** | r |  |  |  |  |  |  |  |  |  |  |  |  | -0.027 |
|  | Sig. |  |  |  |  |  |  |  |  |  |  |  |  | 0.775 |

** Correlation is significant at the 0.01 level (2-tailed).

* Correlation is significant at the 0.05 level (2-tailed).

*r*: correlation coefficient.

pSUM and mSUM mean the total concentration of individual neonicotinoids and individual neonicotinoid metabolites, respectively.

**Table S8** Influent concentrations of NEOs and their metabolites in 21 WWTPs in Shanghai compared with other scenarios (ng/L)

| **Sample** | **Sampling time** | **Location** | **THIA** | **DIN** | **IMI** | **THI** | **CLO** | **ACE** | **NIT** | **IMID** | **∑pNEOs** | **N-DM-ACE** | **DIN-U** | **DN-IMI** | **5-OH-IMI** | **IMI-urea** | **N-DN-THIA** | **∑mNEOs** | **Ref.** |
| --- | --- | --- | --- | --- | --- | --- | --- | --- | --- | --- | --- | --- | --- | --- | --- | --- | --- | --- | --- |
| WWTP influent | October 2022 | Shanghai | 25.17 | 117.89 | 56.45 | 4.56 | 51.57 | 8.49 | 370.83 | 67.60 | 568.17 | 1.78 | 182.37 | 567.36 | 16.02 | 31.23 | 5.95 | 478.20 | This study |
|  | October 2011 | Spain |  |  | 34.44 |  |  |  |  |  | 34.44 |  |  |  |  |  |  |  | (Campo et al., 2013) |
|  | December 2014 | United States |  |  | 60.5 |  | 149.7 | 2.9 |  |  | 213.10 |  |  |  |  |  |  |  | (Sadaria et al., 2016) |
|  | February to March 2022 | Liaocheng | 15.8 | 13.1 | 51.2 |  | 8.48 | 19.8 | 0.77 |  | 109.15 | 11.6 |  |  |  | 266 |  | 277.6 | (Yang et al., 2025) |
|  |  | Hefei | 5.35 | 115 | 11.9 |  | 0.76 | 9.64 | 0.32 |  | 142.97 | 17.5 |  |  |  | 4.4 |  | 21.9 |  |
|  |  | Beijing | 3.15 | 386 | 36.5 |  | 14.3 | 31.2 | 1.65 |  | 472.8 | 25.3 |  |  |  | 2.16 |  | 27.46 |  |
|  |  | Changsha | 5.95 | 48.4 | 504 |  | 3.87 | 2.71 | 0.034 |  | 564.96 | 21.5 |  |  |  | 506 |  | 527.5 |  |
|  |  | Lijiang | 81.7 | 357 | 126 |  | 74.0 | 36.9 | 0.86 |  | 676.46 | 94.6 |  |  |  | 25.9 |  | 120.5 |  |
|  |  | Qingdao | 42.0 | 2146 | 6.66 | 0.1 | 18.7 | 5.45 | 1.23 |  | 2220.14 | 19.9 |  |  |  | 14.1 |  | 34 |  |
|  | 2022-2023 | Yangtze River Delta Industrial WWTP | 32.93 | 45.17 | 37.08 | 24.10 | 85.57 | 13.76 | 176.91 | 75.52 | 491.04 | 0.91 | 17.66 | 42.53 | 3.11 | 13.29 | 9.46 | 86.97 | (Qu et al., 2024) |
|  |  | Yangtze River Delta Municipal WWTP | 30.40 | 59.99 | 54.54 | 7.08 | 15.38 | 17.86 | 495.24 | 14.75 | 695.24 | 12.03 | 69.16 | 167.78 | 0.001 | 33.93 | 24.43 | 307.33 |  |
| Surface water |  | Zhejiang | 26.53 | 119.30 | 38.77 |  | 5.25 | 11.75 | 87.63 | 1.83 | 291.06 |  |  |  |  |  |  |  | (Chen et al., 2024) |
|  | Spring and summer 2015 | Central Yangtze river | 4.29 |  | 6.11 | 0.03 | 0.4 | 2.7 | 0.46 |  | 13.99 | 0.24 |  |  |  |  |  | 0.24 | (Mahai et al., 2019) |
|  | July 2019 | Vietnam | 0.81 |  | 1.93 |  | -- | 5.37 |  |  | 8.11 | 6.4 |  | 2.65 |  | 2.47 |  | 11.52 | (Wan et al., 2021) |
| Drinking water | June 2019 | 32 provinces, China | 6.94 | 0.66 | 5.23 | 0.08 | 3.10 | 1.59 | 0.05 | 0.01 | 17.66 | 0.17 |  | 1.03 |  | 1.75 |  | 2.95 | (Mahai et al., 2021) |
| Groundwater |  |  | 9.09 | 0.23 | 1.88 | 0.03 | 2.11 | 0.22 | 0.03 | 0.002 | 13.59 | 0.34 |  | 0.38 |  | 0.73 |  | 1.45 |  |
| Soil |  | Zhejiang | 7.82 | 9.50 | 49.56 | 1.10 | 6.62 | 2.80 | 2.93 | 0.64 | 80.97 |  |  |  |  |  |  |  | (Chen et al., 2024) |

**Table S9** Ecological risks resulted from NEO point-source emissions to receiving river

| **Compounds** | **MEC (ng/L)** | **RQ** | **Risk level*** |
| --- | --- | --- | --- |
| ACE | 8.49 | 0.14 | Low |
| CLO | 51.57 | 0.92 | Low |
| DIN | 117.89 | 0.18 | Low |
| IMI | 56.45 | 7.31 | Moderate |
| IMID | 67.60 | NA | NA |
| NIT | 370.83 | NA | NA |
| THI | 4.56 | 0.12 | Low |
| THIA | 25.17 | 0.19 | Low |
| N-DM-ACE | 1.78 | NA | NA |
| DN-IMI | 567.36 | NA | NA |
| 5-OH-IMI | 16.02 | NA | NA |
| IMI-urea | 31.23 | NA | NA |
| N-DN-THIA | 5.95 | NA | NA |
| DIN-U | 182.37 | NA | NA |

***** negligible risk: RQ ≤ 0.1; low risk: 0.1 ≤ RQ < 1; moderate risk: 1 ≤ RQ < 10; high risk: RQ ≥ 10.

**Table S10** Park green areas and NEO levels in WWTP influents in 5 districts (Shanghai Municipal Bureau of Statistics, 2022)

| District | Park green area in 2021 (ha.) | pNEOs (ng/L) | mNEOs (ng/L) | NEOs (ng/L) |
| --- | --- | --- | --- | --- |
| FX | 992.44 | 545.86 | 556.45 | 1102.32 |
| JS | 757.62 | 567.57 | 516.12 | 1083.69 |
| MH | 2836.63 | 768.34 | 654.71 | 1423.04 |
| QP | 1258.45 | 485.97 | 402.62 | 888.58 |
| SJ | 1315.56 | 662.23 | 448.90 | 1111.14 |

**Table S11** Annual emission estimates of NEOs from 21 WWTPs to receiving water bodies in suburban Shanghai

|  | **pNEOs** | | | | | | **mNEOs** | | | | | |
| --- | --- | --- | --- | --- | --- | --- | --- | --- | --- | --- | --- | --- |
|  | **Total conc.** **(ng/L)** | **α** | **β** | **ε** | **RR (%)** | **Annual emissions (kg)** | **Total conc. (ng/L)** | **α** | **β** | **ε** | **RR (%)** | **Annual emissions (kg)** |
| FXW-1 | 675.44±365.95 | 5.71 | 498.78 |  | 45.37 | 16.16±8.78 | 1037.20±131.64 | -1.56 | 788.92 |  | 45.37 | 24.82±3.15 |
| FXW-2 | 242.40±78.43 | 5.71 | 498.78 |  | 44.66 | 2.18±0.71 | 527.56±32.82 | -1.56 | 788.92 |  | 44.66 | 4.74±0.30 |
| FXW-3 | 1066.70±955.93 | 5.71 | 498.78 | -3.26 | 45.39 | 42.54±38.14 | 625.46±558.68 | -1.56 | 788.92 | -7.64 | 45.39 | 24.94±22.33 |
| JSW-1 | 504.38±821.31 | 5.71 | 498.78 | -28.67 | 47.91 | 2.69±4.36 | 1229.51±687.59 | -1.56 | 788.92 |  | 47.91 | 6.55±3.67 |
| JSW-2 | 1459.98±546.60 | 5.71 | 498.78 |  | 45.37 | 5.82±2.18 | 950.57±92.87 | -1.56 | 788.92 |  | 45.37 | 3.79±0.37 |
| JSW-3 | 170.97±27.42 | 5.71 | 498.78 |  | 43.40 | 0.71±0.11 | 622.71±10.67 | -1.56 | 788.92 |  | 43.40 | 2.57±0.04 |
| JSW-4 | 355.25±38.80 | 5.71 | 498.78 |  | 47.91 | 4.05±0.44 | 510.38±3.20 | -1.56 | 788.92 |  | 47.91 | 5.82±0.03 |
| JSW-5 | 1174.96±357.43 | 5.71 | 498.78 |  | 25.97 | 31.75±9.66 | 768.07±191.01 | -1.56 | 788.92 | -10.62 | 25.97 | 20.75±5.17 |
| JSW-6 | 792.17±200.11 | 5.71 | 498.78 |  | 25.97 | 16.05±4.06 | 1207.86±199.08 | -1.56 | 788.92 |  | 25.97 | 24.48±4.03 |
| JSW-7 | 667.52±365.87 | 5.71 | 498.78 | 62.87 | 45.37 | 6.66±3.65 | 905.18±111.86 | -1.56 | 788.92 |  | 45.37 | 9.02±1.11 |
| MHW-13 | 777.57±126.83 | 5.71 | 498.78 |  | 47.91 | 29.57±4.84 | 750.18±899.37 | -1.56 | 788.92 |  | 47.91 | 28.53±3.39 |
| QPW-1 | 625.34±138.31 | 5.71 | 498.78 | 50.60 | 47.91 | 6.54±1.45 | 661.96±50.00 | -1.56 | 788.92 |  | 47.91 | 6.92±0.52 |
| QPW-2 | 490.96±217.56 | 5.71 | 498.78 |  | 45.37 | 17.62±7.83 | 689.46±48.71 | -1.56 | 788.92 | -1.29 | 45.37 | 24.75±1.74 |
| QPW-3 | 810.83±161.36 | 5.71 | 498.78 |  | 45.37 | 8.25±1.64 | 719.91±63.12 | -1.56 | 788.92 |  | 45.37 | 7.32±0.64 |
| QPW-4 | 340.74±110.58 | 5.71 | 498.78 |  | 47.91 | 0.16±0.05 | 790.61±28.60 | -1.56 | 788.92 | 3.24 | 47.91 | 0.38±0.01 |
| QPW-5 | 753.27±187.83 | 5.71 | 498.78 |  | 47.91 | 2.86±0.71 | 605.85±45.77 | -1.56 | 788.92 |  | 47.91 | 2.30±0.17 |
| QPW-6 | 450.49±175.77 | 5.71 | 498.78 | -59.72 | 45.37 | 0.45±0.18 | 679.76±199.42 | -1.56 | 788.92 |  | 45.37 | 0.68±0.20 |
| SJW-1 | 796.41±195.69 | 5.71 | 498.78 | -21.83 | 45.37 | 22.23±5.47 | 718.08±29.87 | -1.56 | 788.92 | 16.31 | 45.37 | 20.05±0.83 |
| SJW-2 | 822.45±331.02 | 5.71 | 498.78 |  | 45.37 | 31.98±12.91 | 769.63±75.28 | -1.56 | 788.92 |  | 45.37 | 29.93±2.92 |
| SJW-3 | 573.10±75.40 | 5.71 | 498.78 |  | 45.37 | 15.77±2.08 | 742.33±79.27 | -1.56 | 788.92 |  | 45.37 | 20.43±2.18 |
| SJW-4 | 664.37±278.78 | 5.71 | 498.78 |  | 45.37 | 0.53±0.22 | 710.24±32.83 | -1.56 | 788.92 |  | 45.37 | 0.57±0.03 |
| 21 WWTPs in this study |  |  |  |  |  | 264.57±44.39 |  |  |  |  |  | 269.34±24.41 |

**Table S12** Annual emission estimates of NEOs from all other WWTPs to receiving water bodies in Shanghai

|  | **Essential Information** | | **pNEOs** | | | | | | **mNEOs** | | | | | |
| --- | --- | --- | --- | --- | --- | --- | --- | --- | --- | --- | --- | --- | --- | --- |
|  | **Design capacity**  **(10,000 t/a)** | **Service population**  **(10,000 people)** | **Total conc. (ng/L)** | **α** | **β** | **ε** | **RR (%)** | **Annual emissions (kg)** | **Total conc. (ng/L)** | **α** | **β** | **ε** | **RR (%)** | **Annual emissions (kg)** |
| QPW-7 | 1277.5 | 25 | 641.57±249.45 | 5.71 | 498.78 | -0.003 | 45.37 | 4.48±1.74 | 749.97±83.74 | -1.56 | 788.92 | 0.002 | 45.37 | 5.23±0.58 |
| QPW-8 | 2190 | 28.8 | 663.28±257.76 | 5.71 | 498.78 | -0.003 | 45.37 | 7.94±3.08 | 744.05±82.30 | -1.56 | 788.92 | 0.002 | 45.37 | 8.90±0.99 |
| QPW-9 | 657 | 3.7 | 519.91±202.86 | 5.71 | 498.78 | -0.003 | 45.37 | 1.87±0.73 | 783.16±91.82 | -1.56 | 788.92 | 0.002 | 45.37 | 2.81±0.33 |
| QPW-10 | 91.25 | 1 | 504.49±196.96 | 5.71 | 498.78 | -0.003 | 47.91 | 0.24±0.09 | 787.36±92.84 | -1.56 | 788.92 | 0.002 | 47.91 | 0.37±0.04 |
| SJW-5 | 3650 | 50 | 784.37±304.12 | 5.71 | 498.78 | -0.003 | 45.37 | 15.64±6.05 | 711.01±74.27 | -1.56 | 788.92 | 0.002 | 45.37 | 14.18±1.48 |
| SJW-6 | 620.5 | 6 | 533.05±207.89 | 5.71 | 498.78 | -0.003 | 40.06 | 1.98±0.77 | 779.57±90.95 | -1.56 | 788.92 | 0.002 | 40.06 | 2.90±0.34 |
| SJW-7 | 438 | 6 | 533.05±207.89 | 5.71 | 498.78 | -0.003 | 40.06 | 1.40±0.54 | 779.57±90.95 | -1.56 | 788.92 | 0.002 | 40.06 | 2.05±0.24 |
| BSW-1 | 14600 | 100 | 1069.96±413.48 | 5.71 | 498.78 | -0.003 | 47.91 | 81.37±31.38 | 633.10±55.31 | -1.56 | 788.92 | 0.002 | 47.91 | 48.15±4.23 |
| PDW-1 | 102200 | 712 | 1790.37±689.32 | 5.71 | 498.78 | -0.003 | 47.91 | 953.15±366.42 | 553.43±35.92 | -1.56 | 788.92 | 0.002 | 47.91 | 294.63±19.15 |
| PDW-2 | 40150 | 235 | 1841.05±708.73 | 5.71 | 498.78 | -0.003 | 47.91 | 385.05±147.58 | 422.74±4.13 | -1.56 | 788.92 | 0.002 | 47.91 | 88.42±0.87 |
| PDW-3 | 1460 | 13.1 | 573.60±223.42 | 5.71 | 498.78 | -0.003 | 47.91 | 4.36±1.69 | 768.51±88.26 | -1.56 | 788.92 | 0.002 | 47.91 | 5.84±0.67 |
| PDW-4 | 14600 | 70 | 898.60±347.87 | 5.71 | 498.78 | -0.003 | 45.37 | 71.68±27.79 | 679.85±66.68 | -1.56 | 788.92 | 0.002 | 45.37 | 54.23±5.33 |
| PDW-5 | 40150 | 328.56 | 2375.45±913.35 | 5.71 | 498.78 | -0.003 | 47.91 | 496.82±191.21 | 276.96±31.34 | -1.56 | 788.92 | 0.002 | 47.91 | 57.93±6.54 |
| PDW-6 | 7300 | 79 | 950.01±367.55 | 5.71 | 498.78 | -0.003 | 45.37 | 37.89±14.73 | 665.82±63.27 | -1.56 | 788.92 | 0.002 | 45.37 | 26.55±2.52 |
| PDW-7 | 7300 | 60 | 841.49±325.99 | 5.71 | 498.78 | -0.003 | 45.37 | 33.56±13.00 | 695.43±70.48 | -1.56 | 788.92 | 0.002 | 45.37 | 27.73±2.82 |
| PDW-8 | 43800 | 308.16 | 2258.94±868.74 | 5.71 | 498.78 | -0.003 | 47.91 | 515.40±197.21 | 308.74±23.61 | -1.56 | 788.92 | 0.002 | 47.91 | 70.44±5.38 |
| JDW-1 | 3650 | 8.8 | 555.90±216.64 | 5.71 | 498.78 | -0.003 | 45.37 | 11.08±4.32 | 773.34±89.43 | -1.56 | 788.92 | 0.002 | 45.37 | 15.42±1.78 |
| JDW-2 | 6387.5 | 60 | 841.49±325.99 | 5.71 | 498.78 | -0.003 | 47.91 | 28.00±10.83 | 695.43±70.48 | -1.56 | 788.92 | 0.002 | 47.91 | 23.14±2.35 |
| JDW-3 | 3650 | 8.8 | 549.04±214.02 | 5.71 | 498.78 | -0.003 | 45.37 | 10.95±4.26 | 775.21±89.89 | -1.56 | 788.92 | 0.002 | 45.37 | 15.46±1.79 |
| JDW-4 | 1825 | 39.7 | 725.54±281.60 | 5.71 | 498.78 | -0.003 | 47.91 | 6.90±2.67 | 727.06±78.17 | -1.56 | 788.92 | 0.002 | 47.91 | 6.91±0.74 |
| CMW-1 | 912.5 | 13 | 573.03±223.20 | 5.71 | 498.78 | -0.003 | 47.91 | 2.72±1.06 | 768.67±88.29 | -1.56 | 788.92 | 0.002 | 47.91 | 3.65±0.42 |
| CMW-2 | 1825 | 27 | 653.00±253.82 | 5.71 | 498.78 | -0.003 | 47.91 | 6.21±2.41 | 746.85±82.99 | -1.56 | 788.92 | 0.002 | 47.91 | 7.10±0.79 |
| CMW-3 | 638.75 | 12.8 | 571.89±222.77 | 5.71 | 498.78 | -0.003 | 47.91 | 1.90±0.74 | 768.98±88.37 | -1.56 | 788.92 | 0.002 | 47.91 | 2.56±0.29 |
| CMW-4 | 465.25 | 10.8 | 560.46±218.39 | 5.71 | 498.78 | -0.003 | 47.91 | 1.36±0.53 | 772.09±89.13 | -1.56 | 788.92 | 0.002 | 47.91 | 1.87±0.22 |
| CMW-5 | 182.25 | 7.5 | 541.62±211.17 | 5.71 | 498.78 | -0.003 | 47.91 | 0.51±0.20 | 777.24±90.38 | -1.56 | 788.92 | 0.002 | 47.91 | 0.74±0.09 |
| All WWTPs in Shanghai |  |  |  |  |  |  |  | 2947.03±485.62 |  |  |  |  |  | 1056.56±33.37 |

**References**

Acero, J.L., Real, F.J., Javier Benitez, F., Matamoros, E., 2019. Degradation of neonicotinoids by UV irradiation: Kinetics and effect of real water constituents. Sep Purif Technol 211, 218–226. https://doi.org/10.1016/j.seppur.2018.09.076

Campo, J., Masiá, A., Blasco, C., Picó, Y., 2013. Occurrence and removal efficiency of pesticides in sewage treatment plants of four Mediterranean River Basins. J Hazard Mater 263, 146–157. https://doi.org/10.1016/j.jhazmat.2013.09.061

Chen, Y., Ling, J., Yu, W., Zhang, L., Wu, R., Yang, D., Qu, J., Jin, H., Tao, Z., Shen, Y., Meng, R., Yu, J., Zheng, Q., Shen, G., Du, W., Sun, H., Zhao, M., 2024. Identification of point and nonpoint emission sources of neonicotinoid pollution in regional surface water. Water Res 248. https://doi.org/10.1016/j.watres.2023.120863

Domínguez, J.R., González, T., Correia, S., 2021a. BDD electrochemical oxidation of neonicotinoid pesticides in natural surface waters. Operational, kinetic and energetic aspects. J Environ Manage 298. https://doi.org/10.1016/j.jenvman.2021.113538

Domínguez, J.R., González, T., Correia, S., Domínguez, E.M., 2021b. Sonochemical degradation of neonicotinoid pesticides in natural surface waters. Influence of operational and environmental conditions. Environ Res 197. https://doi.org/10.1016/j.envres.2021.111021

Gusmaroli, L., Mendoza, E., Petrovic, M., Buttiglieri, G., 2020. How do WWTPs operational parameters affect the removal rates of EU Watch list compounds? Science of the Total Environment. https://doi.org/10.1016/j.scitotenv.2020.136773

Iancu, V.I., Radu, G.L., 2018. Occurrence of neonicotinoids in waste water from the Bucharest treatment plant. Analytical Methods 10, 2691–2700. https://doi.org/10.1039/c8ay00510a

Li, X., Zhao, Q., Li, A., Jia, S., Wang, Z., Zhang, Y., Wang, W., Zhou, Q., Pan, Y., Shi, P., 2022. Spatiotemporal distribution and fates of neonicotinoid insecticides during the urban water cycle in the lower reaches of the Yangtze River, China. Water Res 226. https://doi.org/10.1016/j.watres.2022.119232

Mahai, G., Wan, Y., Xia, W., Wang, A., Shi, L., Qian, X., He, Z., Xu, S., 2021. A nationwide study of occurrence and exposure assessment of neonicotinoid insecticides and their metabolites in drinking water of China. Water Res 189. https://doi.org/10.1016/j.watres.2020.116630

Mahai, G., Wan, Y., Xia, W., Yang, S., He, Z., Xu, S., 2019. Neonicotinoid insecticides in surface water from the central Yangtze River, China. Chemosphere 229, 452–460. https://doi.org/10.1016/j.chemosphere.2019.05.040

Ministry of Ecology and Environment of the People’s Republic of China, 2020. The list of the third batch of open facility units [WWW Document]. https://www.mee.gov.cn/home/ztbd/rdzl/sskf/kfss/shs/qh/.

Qu, J., Jin, H., Chen, T., Yu, W., Yang, D., Zhang, Yunhui, Meng, R., Shen, Y., Tao, Z., Zheng, Y., Li, Y., Zhang, Yanyan, Du, W., Yue, S., Zhao, M., Chen, Y., 2024. Regional neonicotinoid pollution in wastewater treatment plants and point source emissions. ACS ES&T Water. https://doi.org/10.1021/acsestwater.4c00560

Sadaria, A.M., Supowit, S.D., Halden, R.U., 2016. Mass balance assessment for six neonicotinoid insecticides during conventional wastewater and wetland treatment: Nationwide reconnaissance in United States wastewater. Environ Sci Technol 50, 6199–6206. https://doi.org/10.1021/acs.est.6b01032

Shanghai Municipal Bureau of Statistics, 2022. Shanghai statistical yearbook 2022 [WWW Document]. https://tjj.sh.gov.cn/tjnj/20230206/804acea250d44d2187f2e37d2e5d36ba.html.

Shanghai Municipal Bureau of Water Resources, 2022. 2022 Annual report of Shanghai drainage facilities [WWW Document]. https://swj.sh.gov.cn/cmsres/4d/4dca56f51fcc4cdb98e64f058023d8a0/a0b71a2d1059308c01483da3574589ec.pdf.

Wan, Y., Han, Q., Wang, Y., He, Z., 2020. Five degradates of imidacloprid in source water, treated water, and tap water in Wuhan, central China. Science of the Total Environment 741. https://doi.org/10.1016/j.scitotenv.2020.140227

Wan, Y., Tran, T.M., Nguyen, V.T., Wang, A., Wang, J., Kannan, K., 2021. Neonicotinoids, fipronil, chlorpyrifos, carbendazim, chlorotriazines, chlorophenoxy herbicides, bentazon, and selected pesticide transformation products in surface water and drinking water from northern Vietnam. Science of the Total Environment 750. https://doi.org/10.1016/j.scitotenv.2020.141507

Wan, Y., Wang, Y., Xia, W., He, Z., Xu, S., 2019. Neonicotinoids in raw, finished, and tap water from Wuhan, Central China: Assessment of human exposure potential. Science of the Total Environment 675, 513–519. https://doi.org/10.1016/j.scitotenv.2019.04.267

Yang, Y., Zhang, Q., Xiao, Yilin, Xiao, Yu, Gao, H., Zhang, S., Covaci, A., Xia, X., 2025. Urban sewage discharge of neonicotinoids and their transformation products threatens aquatic organisms. Water Res 268. https://doi.org/10.1016/j.watres.2024.122740
